# Integrative analysis of gut microbiota and fecal metabolites in metabolic associated fatty liver disease patients

**DOI:** 10.3389/fmicb.2022.969757

**Published:** 2022-08-22

**Authors:** Lidan Yang, Yuzhao Dai, He He, Zhi Liu, Shenling Liao, Yu Zhang, Ga Liao, Zhenmei An

**Affiliations:** ^1^Department of Laboratory Medicine, West China Hospital, Sichuan University, Chengdu, China; ^2^Department of Endocrinology and Metabolism, West China Hospital, Sichuan University, Chengdu, China; ^3^State Key Laboratory of Oral Diseases, National Clinical Research Center for Oral Diseases, West China Hospital of Stomatology, Sichuan University, Chengdu, China; ^4^Department of Information Management, Department of Stomatology Informatics, West China Hospital of Stomatology, Sichuan University, Chengdu, China

**Keywords:** metabolic associated fatty liver disease, intestinal microflora, metabolomics, non-alcoholic fatty liver disease, lipid metabolites

## Abstract

**Objective:**

Metabolic associated fatty liver disease (MAFLD) affects nearly a quarter of the world’s population. Our study aimed to characterize the gut microbiome and overall changes in the fecal and serum metabolomes in MAFLD patients.

**Methods:**

Thirty-two patients diagnosed with MAFLD and 30 healthy individuals (control group, CG) were included in this study, the basic clinical characteristics and laboratory test results including routine biochemistry, etc. were recorded for all, and their serum and fecal samples were collected. A portion of the fecal samples was subjected to 16S rDNA sequencing, and the other portion of the fecal samples and serum samples were subjected to non-targeted metabolomic detection based on liquid chromatography-mass spectrometry (LC–MS). Statistical analysis of clinical data was performed using SPSS software package version 25.0 (SPSS Inc., Chicago, IL, United States). The analysis of 16S rDNA sequencing results was mainly performed by R software (V. 2.15.3), and the metabolomics data analysis was mainly performed by CD 3.1 software. Two-tailed *p* value < 0.05 was considered statistically significant.

**Results:**

The 16S sequencing data suggested that the species richness and diversity of MAFLD patients were reduced compared with controls. At the phylum level, the relative abundance of *Bacteroidota*, *Pseudomonadota*, and *Fusobacteriota* increased and *Bacillota* decreased in MAFLD patients. At the genus level, the relative abundances of *Prevotella*, *Bacteroides*, *Escherichia-Shigella*, etc. increased. 2,770 metabolites were detected in stool samples and 1,245 metabolites were detected in serum samples. The proportion of differential lipid metabolites in serum (49%) was higher than that in feces (21%). There were 22 differential metabolites shared in feces and serum. And the association analysis indicated that LPC 18:0 was positively correlated with *Christensenellaceae_R-7_group*, *Oscillospiraceae_UCG-002*; neohesperidin was also positively correlated with *Peptoniphilus*, *Phycicoccus*, and *Stomatobaculum*.

**Conclusion:**

Microbial sequencing data suggested decreased species richness and diversity and altered β-diversity in feces. Metabolomic analysis identified overall changes in fecal and serum metabolites dominated by lipid molecules. And the association analysis with gut microbes provided potentially pivotal gut microbiota-metabolite combinations in MAFLD patients, which might provide new clues for further research on the disease mechanism and the development of new diagnostic markers and treatments.

## Introduction

Metabolic associated fatty liver disease (MAFLD), a new definition of fatty liver officially proposed by an international expert group in 2020 after the non-alcoholic fatty liver disease (NAFLD; Eslam et al., 2020a), affects at least one-quarter of the adult population worldwide (Powell et al., 2021). MAFLD is closely associated with metabolic disease and its complications, and it has rapidly become one of the leading causes of hepatocellular carcinoma and cirrhosis in Western countries (Younossi et al., 2019). The main complication causing death in patients with MAFLD is CVD. However, liver-related complications are more common in patients with advanced fibrosis or cirrhosis and account for the majority of deaths (Lin et al., 2021). The currently widely accepted treatment recommendations for MAFLD are lifestyle changes aimed at weight loss, and there are no drugs approved for the therapy of MAFLD at this stage (Eslam et al., 2020b; Fouad et al., 2022). Therefore, it is crucial to continue to explore the mechanisms associated with MAFLD and develop new treatments.

In the process of hepatic steatosis and its progression to liver inflammation and liver fibrosis, MAFLD involves the interaction of multiple metabolic, environmental, genetic, and microbial factors (Friedman et al., 2018; Lin et al., 2020). Altered gut-liver axis, increased susceptibility to hepatic triglyceride accumulation, altered lipid metabolism, dyslipidemia, and insulin resistance are key components of the pathophysiology of MAFLD. Notably, multiple studies have shown that the gut-liver axis is closely related to metabolic syndrome, obesity, and type 2 diabetes (Leung et al., 2016; Mardinoglu et al., 2019; Yuan et al., 2019). A high-fat, high-sugar diet and a sedentary lifestyle promote adipogenesis and subclinical inflammation in the intestines, adipose tissue, and liver. Furthermore, this metabolic inflammation in adipose tissue and intestine can promote hepatic adipogenesis and aggravate inflammation through cytokines, fatty acids, dysbiosis of gut flora, and gut barrier disruption (Friedman et al., 2018). The intestinal microbiota is considered to be a new metabolic organ involved in the regulation of host metabolism. The association between microbiota and the pathogenesis of MAFLD has placed those small organisms as a critical focus in MAFLD research. However, the relationship between the gut microbiome and metabolism in MAFLD patients has not been established. Therefore, a comprehensive analysis of the gut microbiome and metabolome may help us uncover the complexity of MAFLD.

Here, we performed 16S gut microbiome sequencing and untargeted metabolomics studies in MAFLD patients and healthy volunteers. We revealed the disruption of gut microbiota homeostasis and the changes in fecal and serum metabolism in patients with MAFLD. In addition, we constructed a map showing the correlation of gut microbiota with fecal and blood metabolism, revealing possible key gut microbe-metabolite combinations, and laying a foundation for further study of the disease mechanism of MAFLD.

## Materials and methods

### Subject enrollment

Thirty-two NAFLD patients and 30 healthy volunteers were recruited from July 2019 to February 2020 at the West China Hospital of Sichuan University (Sichuan Province, China). NAFLD patients were newly diagnosed outpatients and were diagnosed according to the clinical diagnostic criteria recommended by the Chinese Association for the Study of Liver Diseases and the American Association for the Study of Liver Diseases (Fan et al., 2011; Chalasani et al., 2018). The detailed inclusion and exclusion criteria of the case and control groups can be found in the Supplementary material. Patients’ basic clinical characteristics and relevant laboratory test results were recorded, including age, sex, height, weight, body mass index (BMI), and routine biochemical test results (TBIL, DBIL, IBIL, ALT, AST, TP, ALB, GLB, fasting glucose, UREA, CREA, eGFR, URIC, TG, CHOL, HDL-C, LDL-C, ALP, and GGT), thyroid function test results (TSH, FT3, and FT4), etc. This study was approved by the Institutional Review Board of West China Hospital, Sichuan University, exempted from informed consent, and conducted following the Declaration of Helsinki.

### Sample processing

Fecal and serum samples from each volunteer were collected on the same day. Volunteers self-collected samples after defecation in the hospital and immediately transferred the samples to a laboratory freezer at −80°C for cryopreservation. Blood samples were collected from fasting venous blood, placed at room temperature to stratify, and centrifuged at 3,000 rpm for 10 min. Serum samples were collected and frozen in a −80 freezer.

DNA extraction from stool samples was performed using the sodium dodecyl sulfate (SDS) method. Before amplification, check the purity and concentration of DNA by electrophoresis, and dilute the sample DNA with sterile water to obtain the amplification template (target concentration was 1 ng/μl). The 16S V3-V4 region was selected as the amplified region in this study (Abellan-Schneyder et al., 2021). The PCR was completed using specific primers with “barcode short sequences” (used to distinguish each sample), buffers that provide GC bases (Phusion® High-Fidelity PCR Master Mix, New England Biolabs), and high-efficiency, high-fidelity enzymes. The samples were mixed in the same volume according to the PCR product concentration. Purification was carried out using 2% agarose gel electrophoresis, and finally, the target band was recovered with a gel recovery kit (Qiagen). The PCR-free library was constructed using the TruSeq® DNA PCR-Free Sample Preparation Kit based on the Illumina Nova sequencing platform, followed by paired-end sequencing (Caporaso et al., 2012). And Qubit and Q-PCR quantitative detection were used to judge whether the library was qualified or not before running on the computer (NovaS eq6000).

For metabolomics sample processing, firstly 100 mg of liquid nitrogen-ground fecal samples were placed in an EP tube, and 500 μl of 80% methanol in water was added. 100 μl of serum sample was placed in an EP tube, and 400 μl of 80% methanol in water was added. Vortex and shake, stand in an ice bath for 5 min, centrifuge at 15,000 rpm and 4°C for 10 min, take a certain amount of supernatant and add mass spectrometry-grade water to dilute to 53% methanol, and place it in a centrifuge tube at 15,000 *g* and centrifuge at 4°C 10 min. The supernatant was collected and analyzed by liquid chromatography-mass spectrometry technology (LC–MS; Alseekh et al., 2021). In addition, an equal volume of samples was taken from each experimental sample and mixed well as a quality control sample for equilibrating the chromatography-mass spectrometry system and monitoring the instrument status, and evaluating the system stability throughout the experimental process. At the same time, a blank sample was set, which was a 53% methanol aqueous solution containing 0.1% formic acid. The pretreatment process was the same as that of the experimental sample and was mainly used to remove background ions.

### Statistical analysis

Statistical analysis of clinical data was performed using the SPSS software package version 25.0 (SPSS Inc., Chicago, IL, United States). The continuous variables were tested for normality first. Variables with homogeneity of normal variance were expressed as mean ± SD, and a *t*-test was used for comparison between groups; variables that were normal but with unequal variance were expressed as mean ± SD, and the Wilcoxon rank-sum test was used for comparison between groups; non-normal variables were expressed as medians (upper and lower quartiles), and the Wilcoxon rank-sum test was used for comparison between groups. Categorical variables were expressed as frequency (percentage), and the chi-square test was used for comparison between groups. Two-tailed value of *p* < 0.05 was considered statistically significant.

The analysis of the results of 16S rDNA sequencing was mainly done using R software (V. 2.15.3). Using Uparse v7.0.1001 software to cluster effective sequences into operational taxonomic units (OTUs) with 97% consistency, and then performed species annotation analysis according to the SILVA132 SSUrRNA database. The data with the least amount of data in the sample were used as the standard to normalize the data to obtain the relative abundance value of the species. Using Qiime software (V. 1.9.1) to calculate the alpha diversity index (including Observed species, Good’s coverage, Chao1, ACE, Shannon, and Simpson index) and beta diversity index (Unifrac distance and Bray-Curtis distance). T-test and Wilcox test were used for inter-group difference analysis of diversity index. Using R software for principal component analysis (PCA) and principal coordinates analysis (PCoA). Finally, R software was used for a routine *t*-test to obtain taxons with significant differences between groups (value of *p* < 0.05); Furtherly, using LEfSe software, taxons with significant differences between the two groups [linear discriminant analysis (LDA) index > 4] were screened.

Data analysis of non-targeted metabolic results used CD 3.1 software, combined with the mzCloud, mzVault, and MassList database for identification and processing to obtain metabolite qualitative and quantitative results. The final identification results were selected from the compounds with a coefficient of variation value of less than 30% in the quality control samples. Compounds were functionally and taxonomically annotated with the KEGG, Human Metabolome Database (HMDB), and LIPID MAPS databases. The partial least squares discriminant analysis model (PLS-DA) was obtained by multivariate statistical analysis. In order to evaluate the reliability of the model, the PLS-DA model of each group was first established, and the model evaluation parameters (R2, Q2) were obtained through 7-fold cross-validation. The closer the values of R2 and Q2 were to 1, the more stable and reliable the model was. Then, the grouping marks of each sample were randomly scrambled, and further modeling and prediction were performed to determine whether the model was “overfitting.” Each modeling corresponded to a set of R2 and Q2 values, and their regression lines were drawn based on the Q2 and R2 values after 200 scrambles and modeling. When R2 was greater than Q2 and the Q2 regression line and the *Y*-axis intercept were less than 0, it could indicate that the model was not “overfitting.” By calculating the variable importance in projection (VIP) value and fold change (FC) of the first principal component, and combining it with a T-test to find differentially expressed metabolites, setting the screening threshold to VIP > 1.0, FC > 1.5, or FC<0.667, and value of *p* < 0.05. The correlation analysis of differential metabolites and differential flora was performed using Pearson correlation analysis. Based on the RandomForest analysis, the genus-level taxons and metabolome data were separately divided into a test set and validation set (7:3), and then the test set was used to build a random forest model. Important taxons or metabolites were screened out according to MeanDecreaseAccuracy and MeanDecreaseGin, and then each model was cross-validated (10-fold) and ROC curves were drawn.

## Results

### Characterization of participants

A total of 30 healthy controls (control group, CG) and 32 MAFLD volunteers (MAFLD group) were included in this study. The two groups had comparable ages [MAFLD group, 38.50 (33.00–51.75) years; CG, 35.33 (32.50–51.25) years], and the difference was not statistically significant. The basic screen of the participants showed that the BMI of the MAFLD group (26.21 ± 3.80) was significantly higher than that of the CG (23.83 ± 3.28), and the difference was statistically significant (*p* < 0.05). Among laboratory indicators, serum AST, ALT, ALP, GGT, fast glucose, TG, and URIC levels in the MAFLD group were higher than those in the CG, and HDL-C was lower than that in the CG, and the differences were statistically significant. In addition, the serum levels of TB, TP, ALB, and CREA in the MAFLD group were higher than those in the CG, and the difference was not statistically significant. We calculated the Fibrosis-4 index (FIB-4) of all people, and the results showed that the results of the MAFLD group [1.25 (0.70–1.92)] were higher than those of the control group [0.65 (0.33–0.85)], but the difference was not statistically significant. In the MAFLD group, FIB-4 < 1.3 and FIB-4 between 1.3 and 2.67 each accounted for 50%. The results of the serum thyroid function test showed that compared with the CG, the MAFLD patients had increased TSH and decreased FT4 and FT3, but the differences were not statistically significant (Supplementary Table S1; Supplementary Figure S1).

### Altered gut microbiota diversity in MAFLD patients

**Figure 1:**
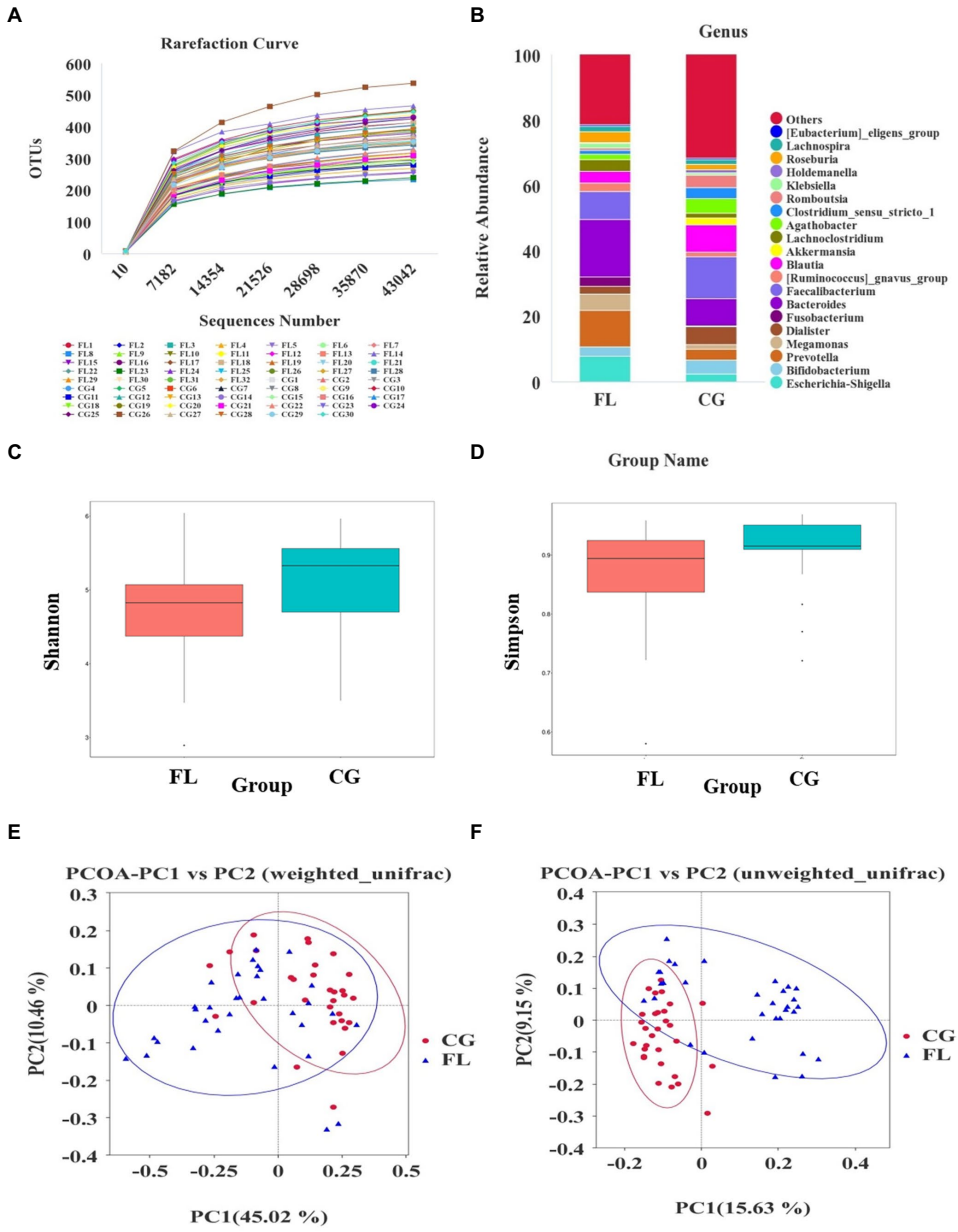
Altered gut microbiota diversity in metabolic associated fatty liver disease (MAFLD) patients. **(A)** Rarefaction curve based on OUT count in control groups (CGs) and MAFLD patients. **(B)** The relative abundance of dominant taxa at the genus level in each group. **(C,D)** The analysis results of alpha diversity indices (Shannon index and Simpson index), both *p* < 0.05. **(E,F)** Principal coordinates analysis (PCoA) analysis based on weighted unifrac distance and unweighted unifrac distance.

An average of 104,138 tags was detected per sample by splicing reads. After quality control, an average of 97,013 pieces of effective data was obtained, and the effective rate of quality control was 61.89%. 1,882 OTUs were obtained by clustering the sequences with 97% identity. According to the rarefaction curve (Figure 1A) and species accumulation boxplot (Supplementary Figure S2A), the current amount of sequencing data and the sample size were reasonable. In addition, the rank abundance curve (Supplementary Figure S2B) and the analysis results of alpha diversity indices (Shannon index, Simpson index, etc.) showed that the species richness and diversity of MAFLD patients were reduced compared with the CG (Figures 1C,D). The difference in beta diversity was observed by PCoA analysis of unifrac distance (Figures 1E,F). In addition, the results of MRPP analysis (*p* < 0.001) and ANOSIM analysis (*p* < 0.001) indicated significant differences in community structure between the MAFLD group and the CG.

**Figure 2:**
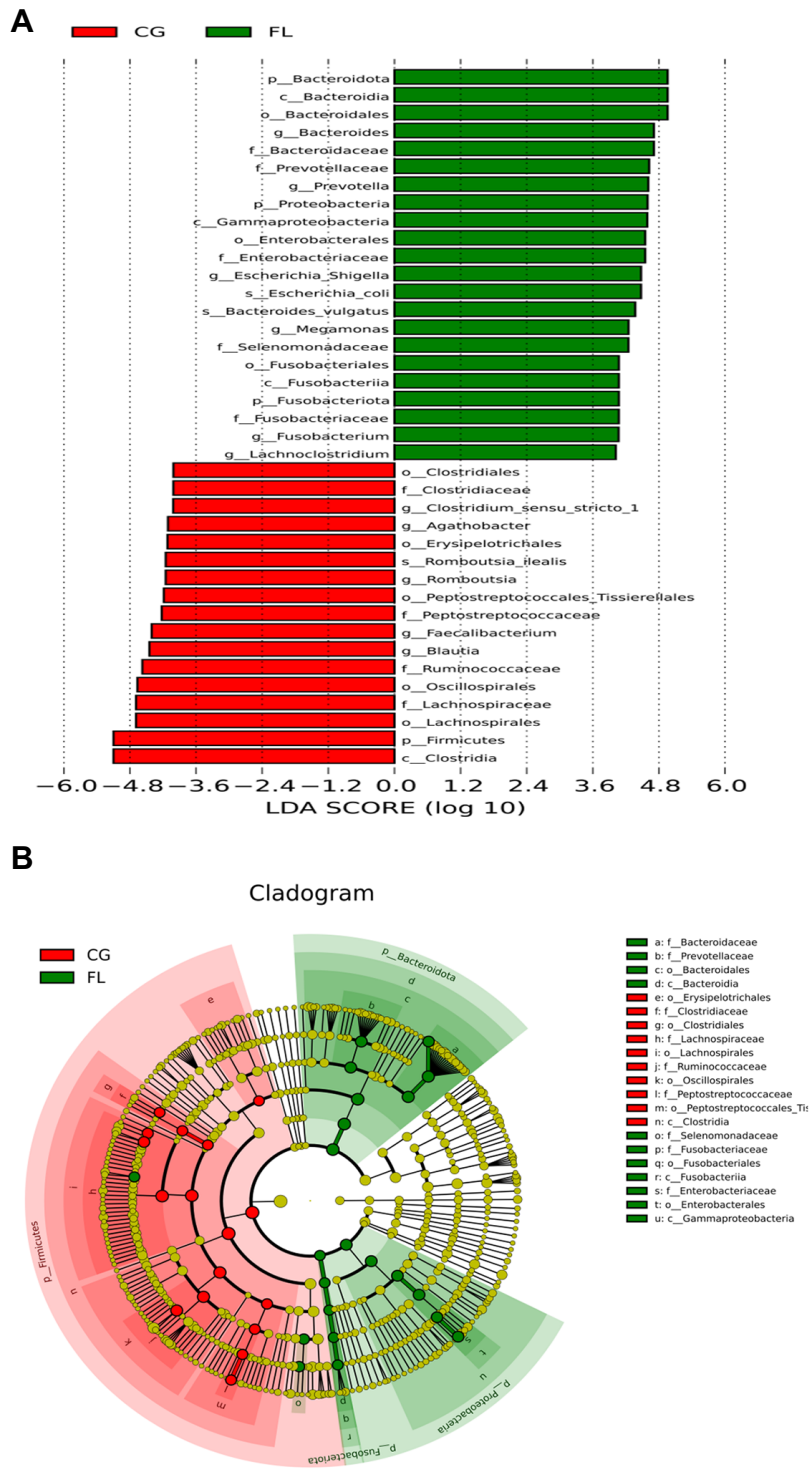
Linear discriminant analysis (LDA) effect size analysis. **(A)** Histogram of the LDA scores for different abundant taxa. Green, enriched in MAFLD patients; red, enriched in CG. **(B)** Cladogram of LEfSe linear discriminant analysis. Red and green circles represent the differences of the most abundant microbiome class. The diameter of each circle is proportional to the relative abundance of the taxon.

The number of OTUs that could be annotated into the database was 1,866 (99.15%). The proportions of annotations at the kingdom level, phylum level, class level, order level, family level, genus level, and species level were 99.15, 91.29, 90.12, 85.44, 79.17, 54.89, and 18.07%, respectively. At the phylum level, we found that the dominant taxa included *Bacillota* (previous name: *Firmicutes*), *Pseudomonadota* (previous name: *Proteobacteria*), *Actinomycetota* (previous name: *Actinobacteria*), and *Bacteroidota* (previous name: *Bacteroidetes*; Supplementary Figure S3A); The dominant genera were *Escherichia-Shigella*, *Bifidobacterium*, and *Prevotella* et al. (Figure 1B); the dominant species were *Escherichia_coli*, *Raoultella_ornithinolytica*, and *Bacteroides_vulgatus* et al. (Supplementary Figure S3B). Through LEfSe analysis, there were 39 taxa (including six grading levels) with LDA value > 4 between the two groups (Figure 2A), and their evolutionary branch diagram was shown in Figure 2B. At the phylum level, the relative abundance of *Bacteroidota*, *Pseudomonadota*, and *Fusobacteriota* increased and *Bacillota* decreased in MAFLD patients. At the genus level, the relative abundances of *Prevotella*, *Bacteroides*, *Escherichia-Shigella*, *Megamonas*, *Fusobacterium*, and *Lachnoclostridium* increased, while *Clostridium_sensu_stricto_1*, *Agathobacter*, *Romboutsia*, *Faecalibacterium*, *Blautia* decreased. Species with increasing relative abundance were *Escherichia_coli*, *Bacteroides_vulgatus*, and species with decreasing relative abundance were *Romboutsia_ilealis*.

### Serum and fecal metabolite profiling in MAFLD patients

A total of 2,770 metabolites were identified in fecal samples, and a total of 1,245 metabolites were identified in serum samples. The classification results of 997 metabolites in fecal samples and 400 in serum samples were obtained through the HMDB (Supplementary Figures S4A,B, S5A,B), of which Lipids and lipid-like molecules were the most classified. Then, we obtained the classification and annotation results of 305 lipid metabolites in fecal samples and 212 in serum samples through the LIPID MAPS database (Supplementary Figures S6A,B, S7A,B), among which Fatty Acids metabolites accounted for the most.

**Figure 3:**
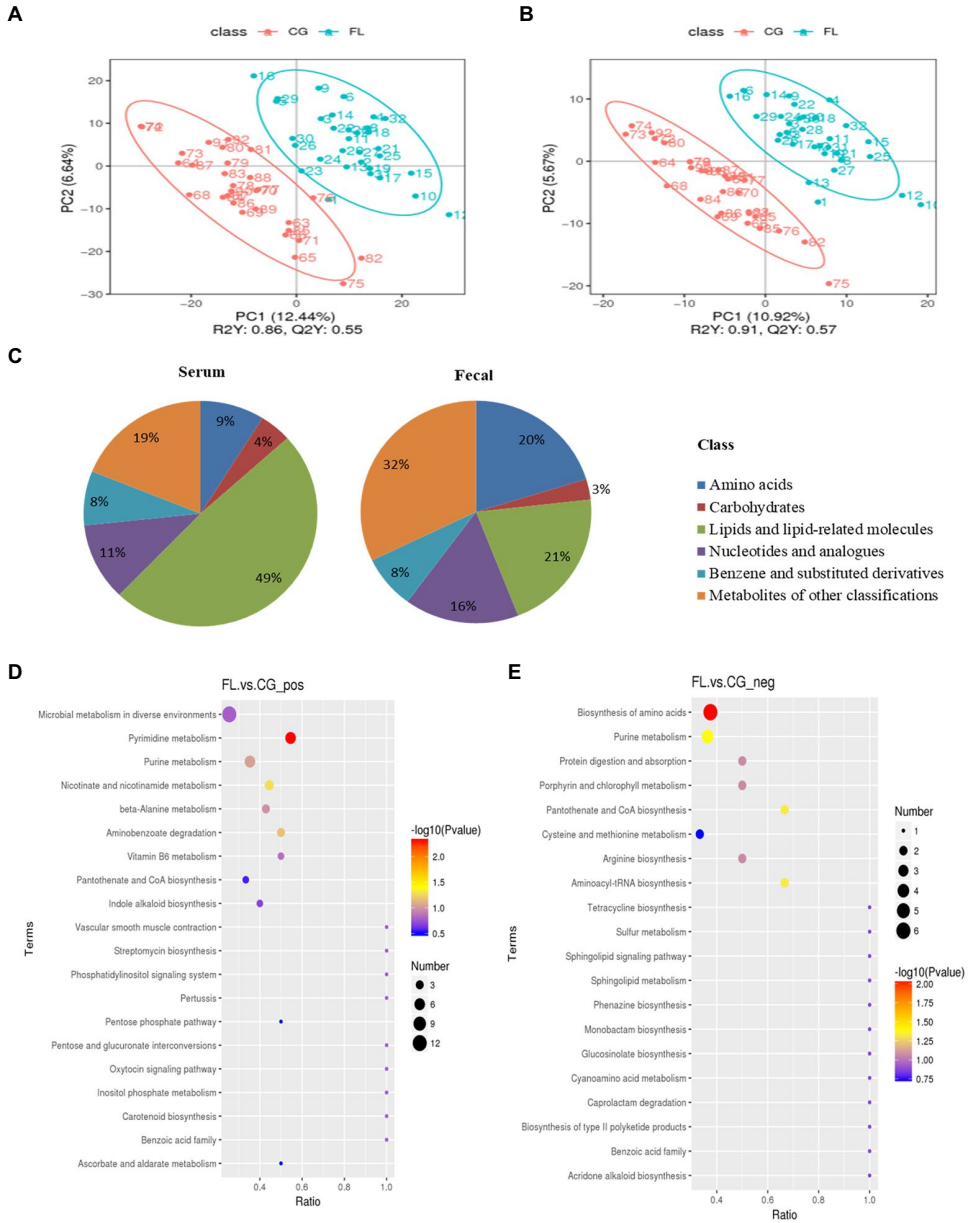
**(A,B)** are the Scatter plot of partial least squares discriminant analysis model (PLS-DA) scores in positive and negative ion mode for fecal metabolites, respectively. The abscissa is the score of the sample on the first principal component, and the ordinate is the score of the sample on the second principal component. R2Y represents the interpretation rate of the model, Q2Y is used to evaluate the predictive ability of the PLS-DA model, and when R2Y is greater than Q2Y, the model is well established. **(C)** The proportion of fecal/serum differential metabolite classification. **(D,E)** are bubble plots of fecal differential metabolite pathway enrichment in positive and negative ion modes, respectively. The abscissa in the figure is the number of differential metabolites in the corresponding metabolic pathway/the total number of metabolites identified in the pathway. The larger the value, the higher the enrichment of differential metabolites in the pathway. The color of the dots represents the value of *p* of the hypergeometric test, and the smaller the value, the greater the reliability of the test. The size of the dots represents the number of differential metabolites in the corresponding pathway.

For differential metabolites screening, we performed PLS-DA on the resulting data (Figures 3A,B; Supplementary Figures S8A,B), and the ranking validation results show that the PLS-DA model was not “overfit” (Supplementary Figures S9A–D). Then, we screened out differential metabolites with VIP > 1.0, FC > 1.5 or FC < 0.667, and value of *p* < 0.05 (Table 1). 34% of differential metabolites in fecal and 40% in serum were classified in HBDM and/or LIPID MAPS database, which mainly included: amino acids, peptides, and analogs; Lipids and lipid-related molecules; Nucleotides and analogs; carbohydrates and carbohydrate conjugates; Benzene and substituted derivatives, etc. Notably, lipids accounted for a large fraction of the significantly variable metabolites in serum and feces, especially in serum, which suggested a disruption of lipid homeostasis in MAFLD patients (Figure 3C). KEGG pathway enrichment results showed that fecal differential metabolites were more enriched in the biosynthesis of amino acids, purine metabolism, pantothenate and CoA biosynthesis, pyrimidine metabolism, nicotinate, and nicotinamide metabolism pathway (Figures 3D,E); serum differential metabolites were more enriched in purine metabolism, pyrimidine metabolism, bile secretion, and pentose phosphate pathway (Supplementary Figures S8C,D). Further analysis found that there were 22 common differential metabolites in feces and serum (Supplementary Table S2), which mainly included purines and purine derivatives: hypoxanthine; amino acids, peptides, and analogues: methionine, gamma-glu-leu, and tyrosylalanine; fatty esters: propionylcarnitine; glycerophosphocholines: lysophosphatidylcholine (LPC 16:0, LPC 18:0); and flavanones: hesperetin and neohesperidin. In particular, during the differential metabolite analysis, we found some bile acids and derivatives: lithocholic acid and taurocholic acid decreased in the serum of MAFLD patients; glycocholic acid increased in the serum of MAFLD patients; and taurodeoxycholic acid, 7-Ketolithocholic acid, allolithocholic acid, and dehydrocholic acid were decreased in the feces of MAFLD patients.

**Table 1:** Metabolite differential screening results.

Sample type	Number of Total Ident.	Number of Total Sig.	Number of Sig. Up	Number of Sig. down
faeces_pos.	1,888	362	47	315
faeces_neg.	882	138	34	104
serum_pos.	731	143	36	117
serum_neg.	414	82	13	69

(1) pos.: positive ion mode; neg.: negative ion mode; (2) Num of Total Ident: Total identification results of metabolites; (3) Num of Total Sig: The total number of metabolites with significant differences; (4) Num of Sig Up: The total number of metabolites significantly upregulated; and (5) Num of Sig down: The total number of metabolites significantly downregulated.

### Correlation between differential bacteria and differential metabolites

Pearson correlation analysis was performed between the top 10 differential bacterial genera in relative abundance and the top 20 differential metabolites in relative abundance, and the differential species-metabolite combinations satisfying |rho| ≥ 0.5 and *p* ≤ 0.05 were screened out (Table 2). Then, we performed a correlation analysis between the common differential metabolites in serum and feces (number = 22) and all differential species in feces (number = 74), and the differential species-metabolite combinations satisfying |rho| ≥ 0.5 and *p* ≤ 0.05 were screened out (Supplementary Table S3). In addition, we correlated the differential bile acids and derivatives with all differential bacterial genera (Supplementary Table S4). Among them, *Erysipelotrichaceae_UCG-003* had a weak positive correlation with serum taurocholic acid (rho = 0.563, *p* < 0.05); allolithocholic acid in feces was associated with *Prevotellaceae_NK3B31_group* (rho = 0.723, *p* < 0.05) and *unidentified_Ruminococcaceae* (rho = 0.797, *p* < 0.05). Finally, we also correlated all differential lipids and lipid-related molecules (number = 44) in serum with fecal differential bacteria (Supplementary Table S5). Among them, the metabolite-genus combinations with strong correlation were: L-Leucyl-L-alanine Hydrate-*Lachnoanaerobaculum* (rho = 0.889, *p* < 0.05), 6-Keto-prostaglandin f1alpha-*Fusicatenibacter* (rho = 0.743, *p* < 0.05), and 6-Keto-prostaglandin f1alpha-*Anaerostipes* (rho = 0.730, *p* < 0.05). The Random Forest analysis results of the genus-level taxons and metabolome data were shown in Supplementary Figures S10–S12.

**Table 2:** Correlation analysis results of fecal differential bacteria and differential metabolites (feces and serum).

	Differential bacteria	Differential metabolites	rho	*p*
Faeces	*Cutibacterium*	Adenosine	0.584	<0.05
	*Intestinibacter*	YMK	0.514	<0.05
	*Intestinibacter*	tert-Butyl N-[1-(aminocarbonyl)- 3-methylbutyl]carbamate	0.562	<0.05
	*Intestinibacter*	L-Alanyl-L-proline	0.521	<0.05
	*Intestinibacter*	3’-Hydroxystanozolol	0.601	<0.05
	*Intestinibacter*	FQH	0.521	<0.05
	*Monoglobus*	tert-Butyl N-[1-(aminocarbonyl)- 3-methylbutyl]carbamate	0.538	<0.05
	*Intestinibacter*	1,5-Anhydro-D-glucitol	0.525	<0.05
	*Intestinibacter*	N-(1-benzothiophen-3-yl)- N′-(1-benzyl-4-piperidinyl)urea	0.560	<0.05
	*Lachnospiraceae*_UCG-004	3’-Dephospho-CoA	0.525	<0.05
Serum	*Neisseria*	Cnidioside A	0.540	<0.05
	*Lachnospiraceae*_FCS020_group	PA (16:0/18:2)	0.503	<0.05
	*Staphylococcus*	LPA 18:2	0.507	<0.05

## Discussion

Evidence accumulated from many preclinical and clinical studies had indicated that the communication between the gut microbiota, its metabolites, and the liver plays a crucial role in the pathogenesis of MAFLD. Here, we recruited 32 patients with MAFLD and investigated overall changes in the gut microbiome in feces and the metabolome in serum and feces. In addition, we identified alterations in several gut microbiota-produced metabolites that may influence the pathogenesis of MAFLD.

The human gut microbiota are mainly composed of four phyla—*Bacteroidota, Bacillota, Pseudomonadota,* and *Actinomycetes*, of which *Bacteroidota* and *Bacillota* dominate the gut (Eckburg et al., 2005; Mokhtari et al., 2017). In the present study, we observed decreased species richness and diversity and altered β-diversity in the feces of MAFLD patients, confirming the development of dysbiosis. Specifically, the relative abundance of *Bacteroidota* and *Pseudomonadota* increased and *Bacillota* decreased in MAFLD patients, which is consistent with previous reports (Boursier et al., 2016; Wang et al., 2016). Under *Bacteroidota*, differential taxa analysis showed that the relative abundance of *Prevotella* and *Bacteroides* increased in MAFLD patients, and the relative abundance of *Bacteroides_vulgatus*, which belonged to *Bacteroides* at the species level, was also increased. Among the increased *Pseudomonadota*, the relative abundances of *Escherichia coli-Shigella* and *Escherichia_coli* increased. Among the reduced *Bacillota*, the taxa with decreased relative abundance at the genus level include: *Clostridium_sensu_stricto_1*, *Agathobacter*, *Faecalibacterium*, *Blautia*, and *Romboutsia*, and the increased ones include: *Megamonas* and *Lachnoclostridium*. The changes in some genera were consistent with the statistical results of a recent meta-analysis that included 1,265 NAFLD patients (from eight countries; Li et al., 2021b). In addition, the relative abundance of *Romboutsia_ilealis* (belonging to *Romboutsia*) also decreased at the species level. In particular, among the differential phyla, we also observed an increase of *Fusobacteriota* in MAFLD patients and an increase of *Fusobacterium* below it.

Previous studies have shown that gut dysbiosis usually led to elevated levels of SCFAs in the gut, with acetate and propionate mainly produced by *Bacteroidota* and butyrate by *Bacillota* (Morrison and Preston, 2016; Feng et al., 2018). Elevated SCFAs promoted the transport of monosaccharides to the liver, while increased hepatic acetate (substrate for fatty acid synthesis) led to the accumulation of triglycerides, and elevated hepatic propionate promoted gluconeogenesis, eventually leading to weight gain (den Besten et al., 2013; Alves-Bezerra and Cohen, 2017). Further, supplementation with SCFAs could also alter the composition of the gut microbiome and prevent the occurrence and progression of NAFLD through multiple mechanisms (Zhou et al., 2017; Zhai et al., 2019; Deng et al., 2020). On the other hand, our study found that the decrease of serum taurocholic acid content was related to *Erysipelotrichaceae_UCG-003*, and the decrease of fecal taurodeoxycholic acid, allolithocholic acid, and dehydrocholic acid content was related to *Subdoligranulum, Prevotellaceae_NK3B31_group,* and *Parvibacter*, respectively. The gut microbiota has a direct impact on bile acid composition and concentration and contributes to NAFLD progression (Ridlon et al., 2014). It was found that NAFLD patients with advanced fibrosis had elevated serum glycocholic acid and fecal deoxycholic acid concentrations, which were associated with increased abundances of *Bacteroidota* and *Lachnospiraceae*, compared with non-NAFLD controls (Adams et al., 2020). Increased secondary bile acid production in the NAFLD gut was associated with *Escherichia* and *Bilophila* (Jiao et al., 2018). *Bacteroides, Bifidobacterium, Clostridium, Lactobacillus,* and *Listeria* can convert bound bile acids to free bile acids *via* bile salt hydrolases, which are subsequently converted to secondary bile acids by *Clostridium* and *Eubacterium* under *Bacillota via* 7αdihydroxylation (Gérard, 2013). Furthermore, *Eggerthella* and *Ruminococcus* were also directly involved in bile acid metabolism (Jia et al., 2018). Thus, our findings suggested that the increase of underlying pathological *Fusobacteriota* and *Pseudomonadota* in MAFLD patients may contribute to the occurrence and development of the disease.

Gut microbiota-related metabolites, such as choline and tryptophan metabolites, SCFAs, bile acids, endogenous ethanol, and lipopolysaccharides, were involved in the pathogenesis of MAFLD (Vallianou et al., 2021). In this study, we performed an overall analysis of fecal and serum metabolites in MAFLD patients, and we identified more metabolites in feces. Although lipid molecules were the most abundant in both, the proportion of differential lipid metabolites in serum (49%) was higher than that in feces (21%), which further confirmed that lipid homeostasis in MAFLD patients was disrupted. At the same time, we also found some other metabolites that may be associated with the pathogenesis of MAFLD. We found that the following metabolites were simultaneously decreased in feces and serum of MAFLD patients: hypoxanthine, propionylcarnitine, tyrosylalanine, hesperetin, methionine, gamma-Glu-Leu, propylparaben, and neohesperidin. However, LPC 16:0, which belongs to glycerophosphocholine, increased in fecal and serum; LPC 18:0 decreased in feces and increased in serum. Studies have shown that the increased concentrations of hypoxanthine and uric acid in hepatocytes contribute to the accumulation of intracellular lipids, which in turn causes the occurrence of oxidative stress associated with the establishment of fatty liver-related diseases, laying the foundation for the development of fibrosis (Stirpe et al., 2002; Taylor et al., 2020). Accumulation of hypoxanthine in the liver established a link between hyperuricemia and NAFLD (Toledo-Ibelles et al., 2021). Hesperetin is a citrus flavonoid found mainly in citrus fruits (oranges, grapefruits, and lemons) with various pharmacological properties, including anticancer, anti-Alzheimer’s disease, and antidiabetic effects (Rekha et al., 2019). Hesperetin can alleviate hepatic steatosis, oxidative stress, inflammatory cell infiltration, and fibrosis in a high-fat diet (HFD)-induced rat model of NAFLD (Li et al., 2021a). Another flavonoid, neohesperidin, can reduce body weight, low-grade inflammation, and insulin resistance by altering the composition of the gut microbiota in mice fed a high-fat diet (Lu et al., 2020). Another study found that neohesperidin enhanced PGC-1α-mediated mitochondrial biosynthesis to alleviate hepatic steatosis in high-fat diet-fed mice (Wang et al., 2020). It was worth noting that our association analysis results suggested that LPC 18:0 was positively correlated with feces *Christensenellaceae_R-7_group*, *Oscillospiraceae_UCG-002*; Propylparaben was correlated with *Erysipelotrichaceae_UCG-003*; neohesperidin was also positively correlated with *Peptoniphilus*, *Phycicoccus*, and *Stomatobaculum* (Supplementary Table S2). However, the discovery and confirmation of the specific role relationship and related mechanisms require further follow-up research. For multi-omics data obtained through designed experiments, the ANOVA simultaneous component analysis (ASCA) and the group-wise ANOVA-simultaneous component analysis (GASCA) were considered to have certain advantages for analyzing the variations ascribable to the main experimental factors and their interactions (Saccenti et al., 2018; Raimondi et al., 2021).

Where we fall short is that due to the inherent worldwide variability in the composition of the gut microbiota (inter-individual and inter-population) it is unclear if our data apply to other areas of the world. Furthermore, previous studies have shown that diet was essential for gut microbial composition and function, and diet, gut microbiome, and metabolome were all interconnected (David et al., 2014; Tang et al., 2019). Although our study excluded patients with “abnormal” dietary habits (e.g., vegetarian food) within the past 12 months, we did not strictly require all participants to adjust their diets but retained their daily dietary habits. Therefore, while our study suggests differences in microbiota and metabolome due to the disease, the study cannot positively tell whether the findings were actually due to disease or diet. Further studies based on the patient’s dietary structure are needed, which may help promote the development of individualized treatments. Finally, there was no significant difference in the FIB-4 index between the MAFLD group [1.25 (0.70–1.92)] and the control group [0.65 (0.33–0.85)], which may be due to the limitation of the sample size. Second, FIB-4 may not be sensitive enough to reflect differences between MAFLD patients and healthy controls when MAFLD patients are in an early stage of the disease. This suggests that we may be able to discover patients with MAFLD in more sensitive ways, such as gut microbiota and metabolites.

In conclusion, the human metabolome consists of interactions of host and microbiota-produced metabolites, and current functional metabolomics studies have focused on determining the role of individual metabolites or individual microbial taxa in MAFLD progression. Characterizing the complex interplay between the gut microbiota, its metabolites, and NAFLD progression remains a challenge. Our data provided a profile of alterations in gut microbes and metabolites in MAFLD patient systems, which may contribute to further studies of MAFLD disease mechanisms and the development of new diagnostic markers and therapeutics.

## Data availability statement

The datasets presented in this study can be found in online repositories. The names of the repository/repositories and accession number(s) can be found below: NCBI—PRJNA851946.

## Ethics statement

The studies involving human participants were reviewed and approved by the Institutional Review Board of West China Hospital, Sichuan University. Written informed consent for participation was not required for this study in accordance with the national legislation and the institutional requirements.

## Author contributions

LY was responsible for the study design, sample collection, data analysis, and manuscript writing. YD participated in the study design and sample collection. HH participated in the study design and manuscript revision. ZL, YZ, and SL participated in the sample collection and data analysis. GL and ZA revised the manuscript. All authors contributed to the article and approved the submitted version.

## Funding

This research was supported by the National Natural Science Foundation of China (NSFC; 32071462) and the Science and Technology Department of Sichuan Province, China (2021YFH0167).

## Conflict of interest

The authors declare that the research was conducted in the absence of any commercial or financial relationships that could be construed as a potential conflict of interest.

## Publisher’s note

All claims expressed in this article are solely those of the authors and do not necessarily represent those of their affiliated organizations, or those of the publisher, the editors and the reviewers. Any product that may be evaluated in this article, or claim that may be made by its manufacturer, is not guaranteed or endorsed by the publisher.

## Supplementary material

The Supplementary material for this article can be found online at: https://www.frontiersin.org/articles/10.3389/fmicb.2022.969757/full#supplementary-material

## References

[ref1] Abellan-Schneyder I. Matchado M. S. Reitmeier S. Sommer A. Sewald Z. Baumbach J.. (2021). Primer, pipelines, parameters: issues in 16S rRNA gene sequencing. mSphere 6, 222–224. doi: 10.1128/mSphere.01202-20, PMID: 33627512 PMC8544895

[ref2] Adams L. A. Wang Z. Liddle C. Melton P. E. Ariff A. Chandraratna H.. (2020). Bile acids associate with specific gut microbiota, low-level alcohol consumption and liver fibrosis in patients with non-alcoholic fatty liver disease. Liver Int. 40, 1356–1365. doi: 10.1111/liv.14453, PMID: 32243703

[ref3] Alseekh S. Aharoni A. Brotman Y. Contrepois K. D’Auria J. Ewald J.. (2021). Mass spectrometry-based metabolomics: a guide for annotation, quantification and best reporting practices. Nat. Methods 18, 747–756. doi: 10.1038/s41592-021-01197-1, PMID: 34239102 PMC8592384

[ref4] Alves-Bezerra M. Cohen D. E. (2017). Triglyceride metabolism in the liver. Compr. Physiol. 8, 1–8. doi: 10.1002/cphy.c170012, PMID: 29357123 PMC6376873

[ref5] Boursier J. Mueller O. Barret M. Machado M. Fizanne L. Araujo-Perez F.. (2016). The severity of nonalcoholic fatty liver disease is associated with gut dysbiosis and shift in the metabolic function of the gut microbiota. Hepatology 63, 764–775. doi: 10.1002/hep.28356, PMID: 26600078 PMC4975935

[ref6] Caporaso J. G. Lauber C. L. Walters W. A. Berg-Lyons D. Huntley J. Fierer N.. (2012). Ultra-high-throughput microbial community analysis on the Illumina HiSeq and MiSeq platforms. ISME J. 6, 1621–1624. doi: 10.1038/ismej.2012.8, PMID: 22402401 PMC3400413

[ref7] Chalasani N. Younossi Z. Lavine J. E. Charlton M. Cusi K. Rinella M.. (2018). The diagnosis and management of nonalcoholic fatty liver disease: practice guidance from the American Association for the Study of Liver Diseases. Hepatology 67, 328–357. doi: 10.1002/hep.29367, PMID: 28714183

[ref8] David L. A. Maurice C. F. Carmody R. N. Gootenberg D. B. Button J. E. Wolfe B. E.. (2014). Diet rapidly and reproducibly alters the human gut microbiome. Nature 505, 559–563. doi: 10.1038/nature12820, PMID: 24336217 PMC3957428

[ref9] den Besten G. Lange K. Havinga R. van Dijk T. H. Gerding A. van Eunen K.. (2013). Gut-derived short-chain fatty acids are vividly assimilated into host carbohydrates and lipids. Am. J. Physiol. Gastrointest. Liver Physiol. 305, G900–G910. doi: 10.1152/ajpgi.00265.2013, PMID: 24136789

[ref10] Deng M. Qu F. Chen L. Liu C. Zhang M. Ren F.. (2020). SCFAs alleviated steatosis and inflammation in mice with NASH induced by MCD. J. Endocrinol. 245, 425–437. doi: 10.1530/JOE-20-0018, PMID: 32302970

[ref11] Eckburg P. B. Bik E. M. Bernstein C. N. Purdom E. Dethlefsen L. Sargent M.. (2005). Diversity of the human intestinal microbial flora. Science 308, 1635–1638. doi: 10.1126/science.1110591, PMID: 15831718 PMC1395357

[ref12] Eslam M. Newsome P. N. Sarin S. K. Anstee Q. M. Targher G. Romero-Gomez M.. (2020a). A new definition for metabolic dysfunction-associated fatty liver disease: an international expert consensus statement. J. Hepatol. 73, 202–209. doi: 10.1016/j.jhep.2020.03.039, PMID: 32278004

[ref13] Eslam M. Sarin S. K. Wong V. W. Fan J. G. Kawaguchi T. Ahn S. H.. (2020b). The Asian Pacific Association for the Study of the liver clinical practice guidelines for the diagnosis and management of metabolic associated fatty liver disease. Hepatol. Int. 14, 889–919. doi: 10.1007/s12072-020-10094-2, PMID: 33006093

[ref14] Fan J. G. Jia J. D. Li Y. M. Wang B. Y. Lu L. G. Shi J. P.. (2011). Guidelines for the diagnosis and management of nonalcoholic fatty liver disease: update 2010: (published in Chinese on Chinese journal of Hepatology 2010; 18:163-166). J. Dig. Dis. 12, 38–44. doi: 10.1111/j.1751-2980.2010.00476.x, PMID: 21276207

[ref15] Feng W. Ao H. Peng C. (2018). Gut microbiota, short-chain fatty acids, and herbal medicines. Front. Pharmacol. 9:1354. doi: 10.3389/fphar.2018.01354, PMID: 30532706 PMC6265305

[ref16] Fouad Y. Esmat G. Elwakil R. Zakaria S. Yosry A. Waked I.. (2022). The egyptian clinical practice guidelines for the diagnosis and management of metabolic associated fatty liver disease. Saudi J. Gastroenterol. 28, 3–20. doi: 10.4103/sjg.sjg_357_21, PMID: 35083973 PMC8919931

[ref17] Friedman S. L. Neuschwander-Tetri B. A. Rinella M. Sanyal A. J. (2018). Mechanisms of NAFLD development and therapeutic strategies. Nat. Med. 24, 908–922. doi: 10.1038/s41591-018-0104-9, PMID: 29967350 PMC6553468

[ref18] Gérard P. (2013). Metabolism of cholesterol and bile acids by the gut microbiota. Pathogens 3, 14–24. doi: 10.3390/pathogens3010014 25437605 PMC4235735

[ref19] Jia W. Xie G. Jia W. (2018). Bile acid-microbiota crosstalk in gastrointestinal inflammation and carcinogenesis. Nat. Rev. Gastroenterol. Hepatol. 15, 111–128. doi: 10.1038/nrgastro.2017.119, PMID: 29018272 PMC5899973

[ref20] Jiao N. Baker S. S. Chapa-Rodriguez A. Liu W. Nugent C. A. Tsompana M.. (2018). Suppressed hepatic bile acid signalling despite elevated production of primary and secondary bile acids in NAFLD. Gut 67, 1881–1891. doi: 10.1136/gutjnl-2017-314307, PMID: 28774887

[ref21] Leung C. Rivera L. Furness J. B. Angus P. W. (2016). The role of the gut microbiota in NAFLD. Nat. Rev. Gastroenterol. Hepatol. 13, 412–425. doi: 10.1038/nrgastro.2016.85 27273168

[ref22] Li J. Wang T. Liu P. Yang F. Wang X. Zheng W.. (2021a). Hesperetin ameliorates hepatic oxidative stress and inflammation via the PI3K/AKT-Nrf2-ARE pathway in oleic acid-induced HepG2 cells and a rat model of high-fat diet-induced NAFLD. Food Funct. 12, 3898–3918. doi: 10.1039/D0FO02736G, PMID: 33977953

[ref23] Li F. Ye J. Shao C. Zhong B. (2021b). Compositional alterations of gut microbiota in nonalcoholic fatty liver disease patients: a systematic review and meta-analysis. Lipids Health Dis. 20:22. doi: 10.1186/s12944-021-01440-w, PMID: 33637088 PMC7908766

[ref24] Lin Y. C. Wu C. C. Ni Y. H. (2020). New perspectives on genetic prediction for pediatric metabolic associated fatty liver disease. Front. Pediatr. 8:603654. doi: 10.3389/fped.2020.603654, PMID: 33363067 PMC7755886

[ref25] Lin H. Zhang X. Li G. Wong G. L. Wong V. W. (2021). Epidemiology and clinical outcomes of metabolic (dysfunction)-associated fatty liver disease. J. Clin. Transl. Hepatol. 9, 972–982. doi: 10.14218/JCTH.2021.00201, PMID: 34966660 PMC8666360

[ref26] Lu J. F. Zhu M. Q. Zhang H. Liu H. Xia B. Wang Y. L.. (2020). Neohesperidin attenuates obesity by altering the composition of the gut microbiota in high-fat diet-fed mice. FASEB J. 34, 12053–12071. doi: 10.1096/fj.201903102RR, PMID: 32729978

[ref27] Mardinoglu A. Ural D. Zeybel M. Yuksel H. H. Uhlén M. Borén J. (2019). The potential use of metabolic cofactors in treatment of NAFLD. Nutrients 11:1578. doi: 10.3390/nu11071578, PMID: 31336926 PMC6682907

[ref28] Mokhtari Z. Gibson D. L. Hekmatdoost A. (2017). Nonalcoholic fatty liver disease, the gut microbiome, and diet. Adv. Nutr. 8, 240–252. doi: 10.3945/an.116.013151, PMID: 28298269 PMC5347097

[ref29] Morrison D. J. Preston T. (2016). Formation of short chain fatty acids by the gut microbiota and their impact on human metabolism. Gut Microbes 7, 189–200. doi: 10.1080/19490976.2015.1134082, PMID: 26963409 PMC4939913

[ref30] Powell E. E. Wong V. W. Rinella M. (2021). Non-alcoholic fatty liver disease. Lancet 397, 2212–2224. doi: 10.1016/S0140-6736(20)32511-3 33894145

[ref31] Raimondi S. Calvini R. Candeliere F. Leonardi A. Ulrici A. Rossi M.. (2021). Multivariate analysis in microbiome description: correlation of human gut protein degraders, metabolites, and predicted metabolic functions. Front. Microbiol. 12:723479. doi: 10.3389/fmicb.2021.723479, PMID: 34603248 PMC8484906

[ref32] Rekha S. S. Pradeepkiran J. A. Bhaskar M. (2019). Bioflavonoid hesperidin possesses the anti-hyperglycemic and hypolipidemic property in STZ induced diabetic myocardial infarction (DMI) in male Wister rats. J. Nutr. Intermed. Metabol. 15, 58–64. doi: 10.1016/j.jnim.2018.12.004

[ref33] Ridlon J. M. Kang D. J. Hylemon P. B. Bajaj J. S. (2014). Bile acids and the gut microbiome. Curr. Opin. Gastroenterol. 30, 332–338. doi: 10.1097/MOG.0000000000000057, PMID: 24625896 PMC4215539

[ref34] Saccenti E. Smilde A. K. Camacho J. (2018). Group-wise ANOVA simultaneous component analysis for designed omics experiments. Metabolomics: official journal of the Metabolomic. Society 14:73. doi: 10.1007/s11306-018-1369-1 PMC5962647 29861703

[ref35] Stirpe F. Ravaioli M. Battelli M. G. Musiani S. Grazi G. L. (2002). Xanthine oxidoreductase activity in human liver disease. Am. J. Gastroenterol. 97, 2079–2085. doi: 10.1111/j.1572-0241.2002.05925.x, PMID: 12190180

[ref36] Tang Z. Z. Chen G. Hong Q. Huang S. Smith H. M. Shah R. D.. (2019). Multi-Omic analysis of the microbiome and metabolome in healthy subjects reveals microbiome-dependent relationships between diet and metabolites. Front. Genet. 10:454. doi: 10.3389/fgene.2019.00454, PMID: 31164901 PMC6534069

[ref37] Taylor R. S. Taylor R. J. Bayliss S. Hagström H. Nasr P. Schattenberg J. M.. (2020). Association Between fibrosis stage and outcomes of patients With nonalcoholic fatty liver disease: a systematic review and meta-analysis. Gastroenterology 158, 1611–25.e12. doi: 10.1053/j.gastro.2020.01.043, PMID: 32027911

[ref38] Toledo-Ibelles P. Gutiérrez-Vidal R. Calixto-Tlacomulco S. Delgado-Coello B. Mas-Oliva J. (2021). Hepatic accumulation of hypoxanthine: a link between Hyperuricemia and nonalcoholic fatty liver disease. Arch. Med. Res. 52, 692–702. doi: 10.1016/j.arcmed.2021.04.005, PMID: 33966916

[ref39] Vallianou N. Christodoulatos G. S. Karampela I. Tsilingiris D. Magkos F. Stratigou T.. (2021). Understanding the role of the gut microbiome and microbial metabolites in non-alcoholic fatty liver disease: current evidence and perspectives. Biomol. Ther. 12:56. doi: 10.3390/biom12010056, PMID: 35053205 PMC8774162

[ref40] Wang B. Jiang X. Cao M. Ge J. Bao Q. Tang L.. (2016). Altered fecal microbiota correlates with liver biochemistry in nonobese patients with non-alcoholic fatty liver disease. Sci. Rep. 6:32002. doi: 10.1038/srep32002, PMID: 27550547 PMC4994089

[ref41] Wang S. W. Sheng H. Bai Y. F. Weng Y. Y. Fan X. Y. Lou L. J.. (2020). Neohesperidin enhances PGC-1α-mediated mitochondrial biogenesis and alleviates hepatic steatosis in high fat diet fed mice. Nutr. Diabetes 10:27. doi: 10.1038/s41387-020-00130-3, PMID: 32759940 PMC7406515

[ref42] Younossi Z. Stepanova M. Ong J. P. Jacobson I. M. Bugianesi E. Duseja A.. (2019). Nonalcoholic steatohepatitis is the fastest growing cause of hepatocellular carcinoma in liver transplant candidates. Clin. Gastroenterol. Hepatol. 17, 748–755.e3. doi: 10.1016/j.cgh.2018.05.057, PMID: 29908364

[ref43] Yuan J. Chen C. Cui J. Lu J. Yan C. Wei X.. (2019). Fatty liver disease caused by high-alcohol-producing *Klebsiella pneumoniae*. Cell Metab. 30, 675–88.e7. doi: 10.1016/j.cmet.2019.08.018, PMID: 31543403

[ref44] Zhai S. Qin S. Li L. Zhu L. Zou Z. Wang L. (2019). Dietary butyrate suppresses inflammation through modulating gut microbiota in high-fat diet-fed mice. FEMS Microbiol. Lett. 366:fnz153. doi: 10.1093/femsle/fnz153, PMID: 31295342

[ref45] Zhou D. Pan Q. Xin F. Z. Zhang R. N. He C. X. Chen G. Y.. (2017). Sodium butyrate attenuates high-fat diet-induced steatohepatitis in mice by improving gut microbiota and gastrointestinal barrier. World J. Gastroenterol. 23, 60–75. doi: 10.3748/wjg.v23.i1.60, PMID: 28104981 PMC5221287

