# Supplementary material for: Integrative analysis of gut microbiota and fecal metabolites in metabolic associated fatty liver disease patients
Source: Front Microbiol. 2022 Aug 22;13:969757. doi: 10.3389/fmicb.2022.969757 (PMC9441872; doi:10.3389/fmicb.2022.969757)
Supplement: Supplementary file 1 [file Data_Sheet_1.docx]

***Supplementary Material***

1. **Case group inclusion and exclusion criteria**

**Inclusion criteria**

1. Age: 18-65 years old;
2. Patients with evidence of hepatic steatosis demonstrated by imaging (ultrasound, FibroScan-CAP, CT, or MRI) or histology and diagnosed as NAFLD by the clinical combination of clinical features and laboratory tests.
3. There is no history of alcohol drinking habit or the ethanol intake per week was less than 140g in men (70g in women) in the past 12 months;
4. Specific diseases that could lead to steatosis, such as viral hepatitis, drug-induced liver disease, total parenteral nutrition, Wilson's disease, and autoimmune liver disease, can be excluded.

**Exclusion criteria**

1. Patients with other chronic gastrointestinal diseases such as IBS, IBD, chronic constipation, celiac disease;
2. Patients with a history of chronic systemic autoimmune diseases with gastrointestinal involvement;
3. Patients with "abnormal" dietary habits (e.g. vegetarian food) within the past 12 months, or those who have been exposed to drugs or interventions such as antibiotics, immunosuppressants, chemotherapy, etc. within the past 3 months that have compromised the composition of the gut microbiome;
4. Patients receiving long-term chronic treatment with proton pump inhibitors;
5. Patients with severe cardiovascular disease, kidney disease, malignant tumor, etc.;
6. Patients with poor blood sugar control and HbA1c>9.5%;
7. Patients with a history of neurological disease and mental illness.
8. **Control group inclusion and exclusion criteria**

**Inclusion criteria**

1. Age: 18-65 years old;
2. Healthy subjects who had no clinical signs or symptoms of illness, including evidence of viral hepatitis and metabolic syndromes, etc.;
3. Imaging evidence that can rule out hepatic steatosis can be found.

**Exclusion criteria**

1. There is no history of alcohol drinking habit or the ethanol intake per week was less than 140g in men (70g in women) in the past 12 months;
2. Those who have had "abnormal" eating habits (eg vegetarian) in the past 12 months, or have been exposed to drugs such as antibiotics that damage the composition of the gut microbiome in the past 3 months.
3. **Supplementary Figures**


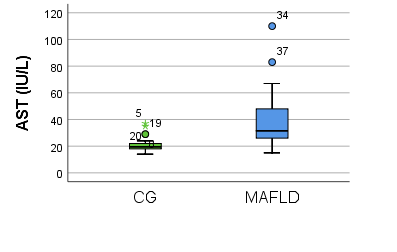

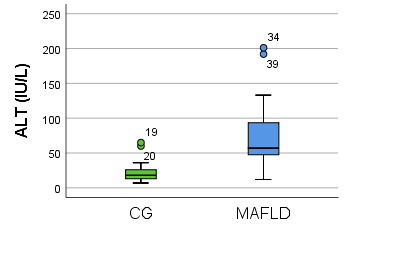

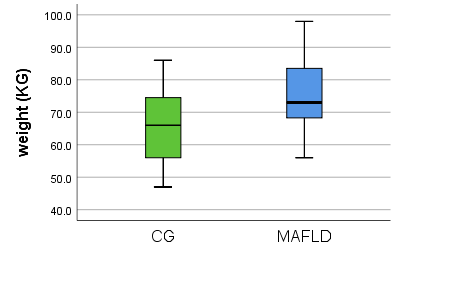

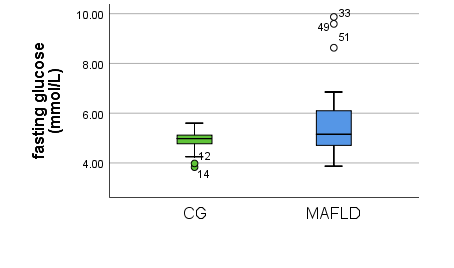

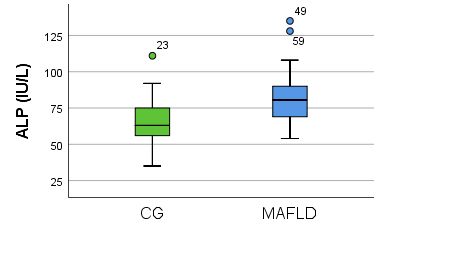

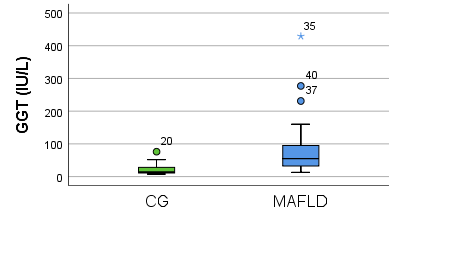

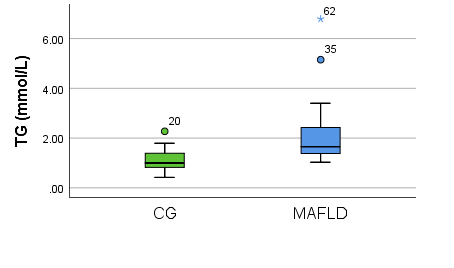

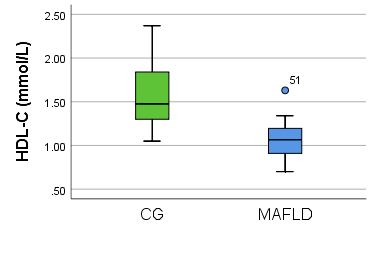

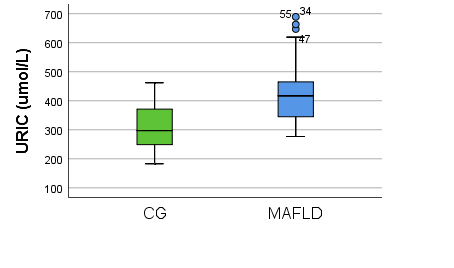


**Fig. S1** Characterization of Participants. ALT: Alanine aminotransferase; AST: Aspartate aminotransferase; ALP: Alkaline phosphatas; GGT: Glycine transpeptidase; TG: triglycerides; HDL-c: High-density lipoprotein-cholesterol; URIC: Uric acid. All p < 0.05.


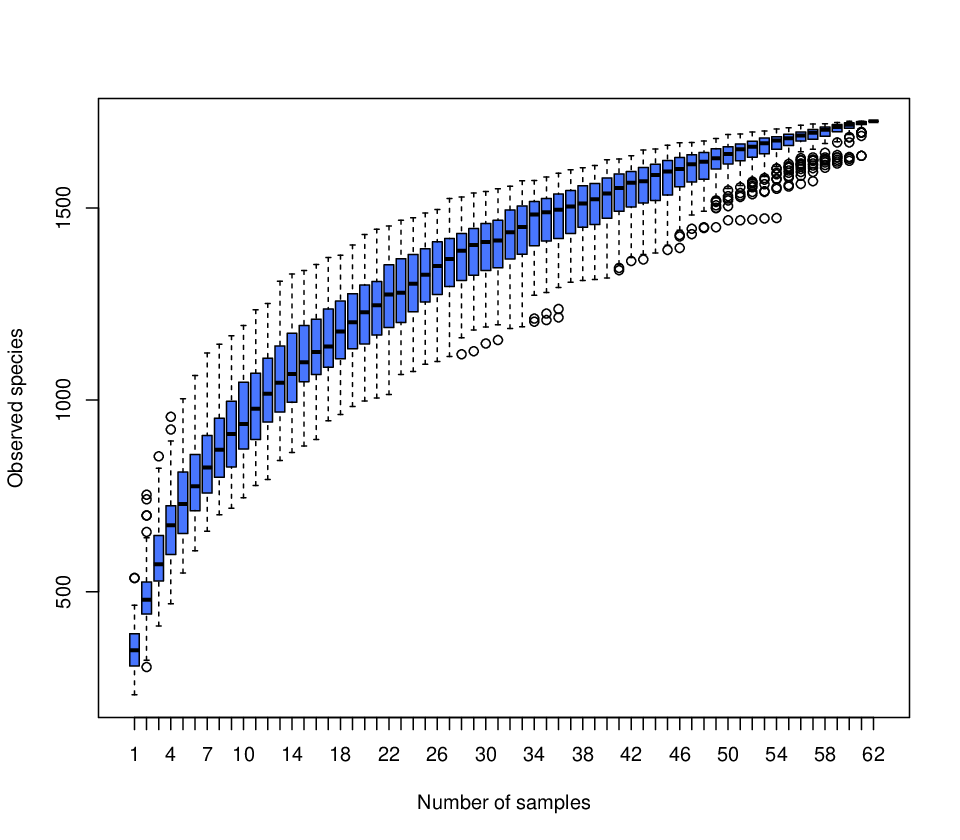


Species accumulation boxplot


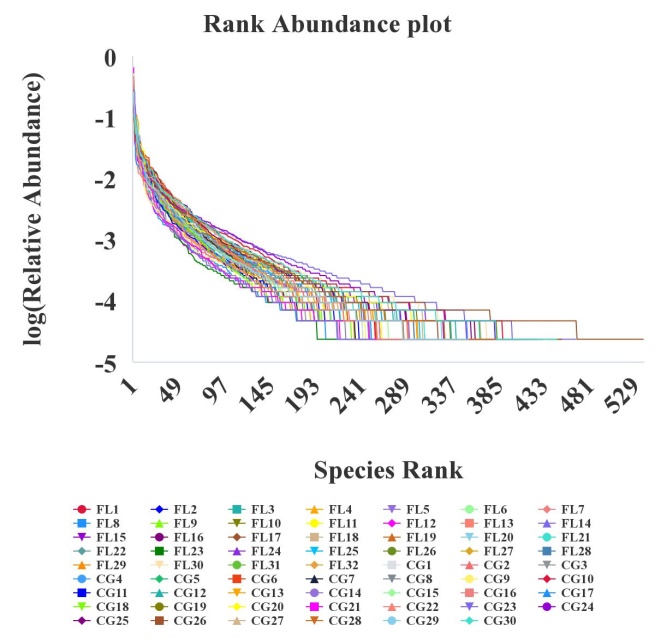


**Fig. S2** (A) In the Rank Abundance curve, the abscissa is the sequence number sorted by the abundance of OTUs, and the ordinate is the relative abundance of the corresponding OTUs. Different samples are represented by polylines with different colors. (B) In the species accumulation boxplot, the abscissa is the sample size; the ordinate is the number of OTUs after sampling. The results reflect the rate at which new OTUs (new species) emerge under continuous sampling.

**A**

**B**


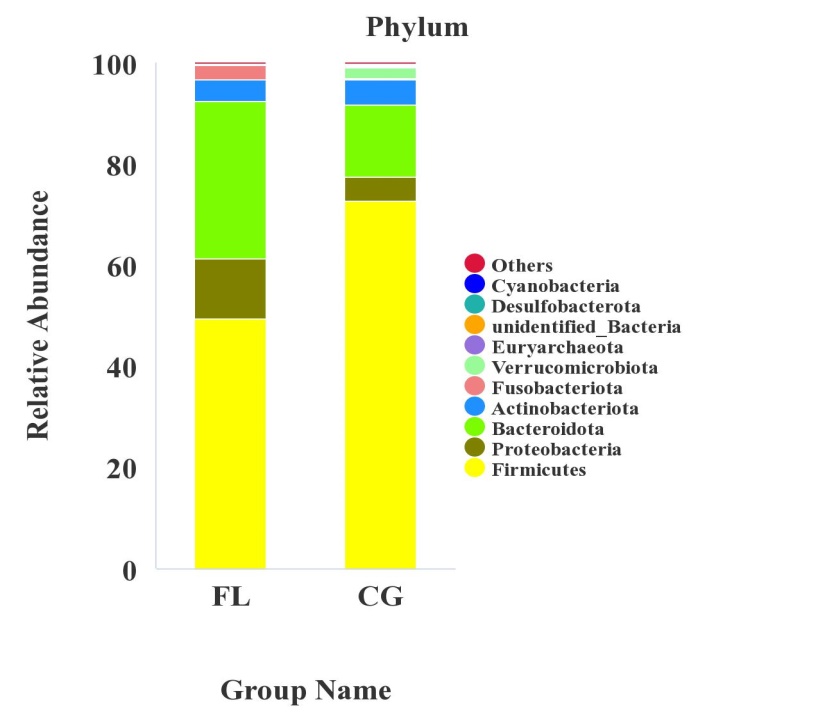

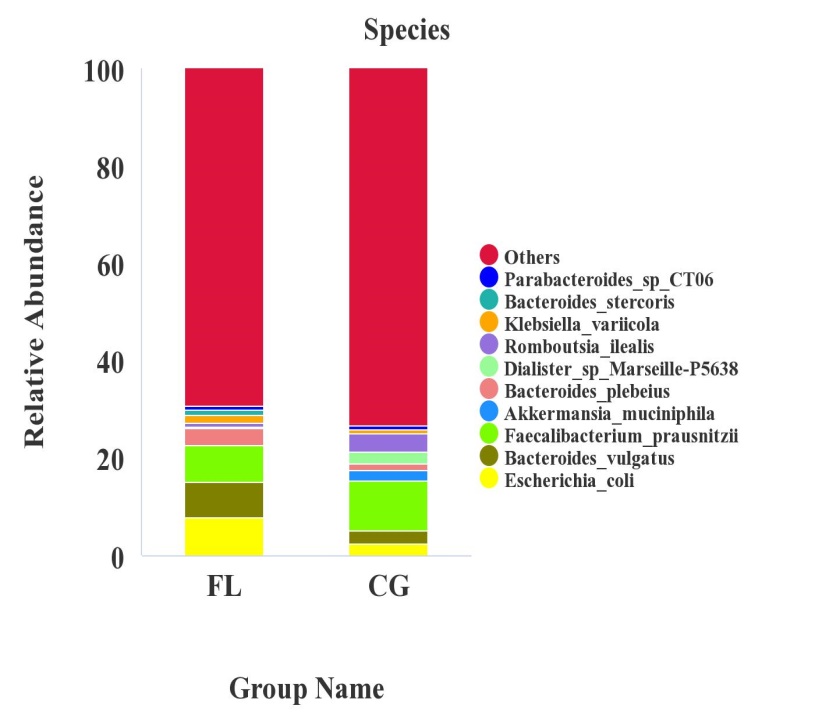


**A**

**B**

**Fig. S3** The relative abundance of dominant taxa at the phylum level (A) and the species level (B) in each group.


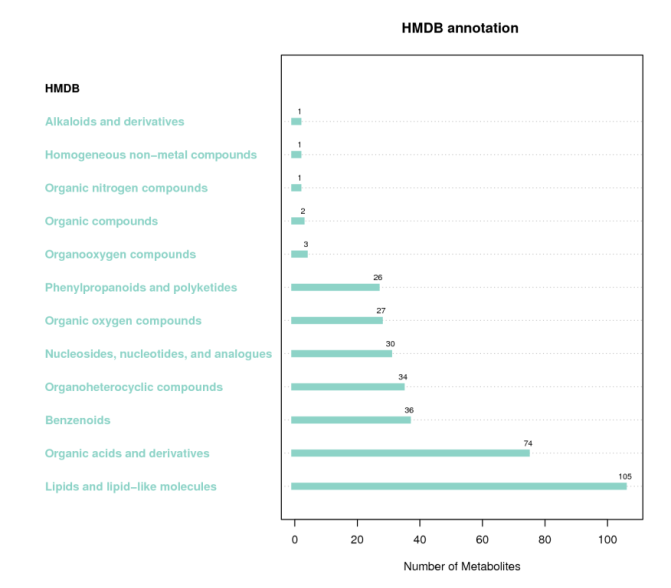


**A**


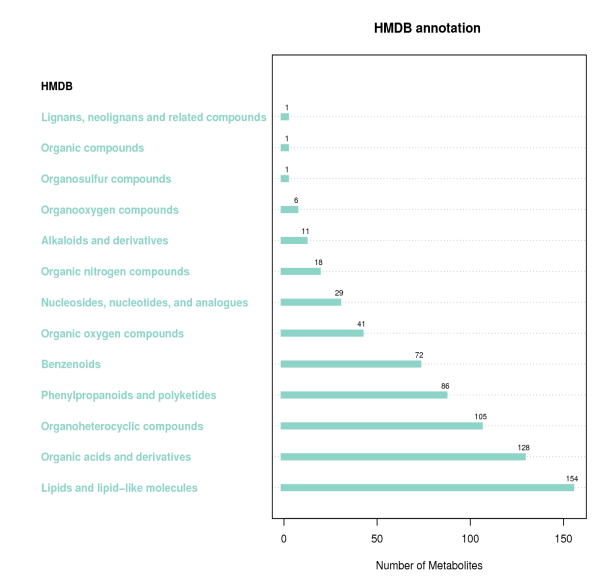


**B**

**Fig. S4** Fecal metabolite classification annotation results in the Super Class of HMDB: (A) negative ion mode (neg.); (B) positive ion mode (pos.).

**A**

**B**

**Fig. S5** Serum metabolite classification annotation results in the Super Class of HMDB: (A) negative ion mode (neg.); (B) positive ion mode (pos.).


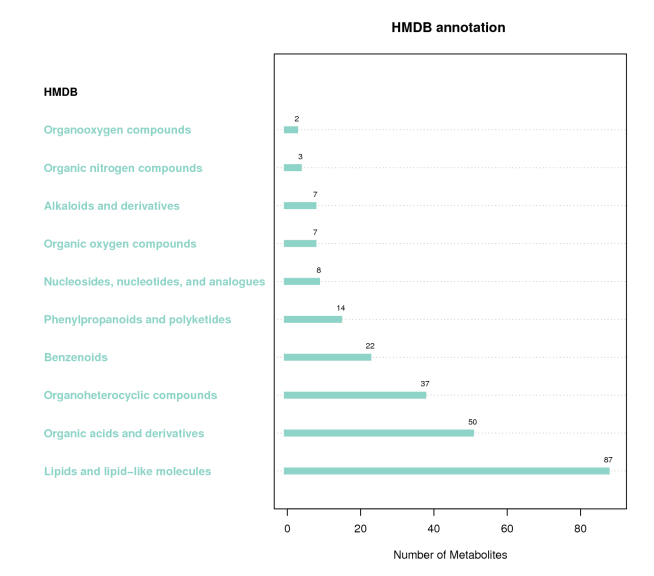

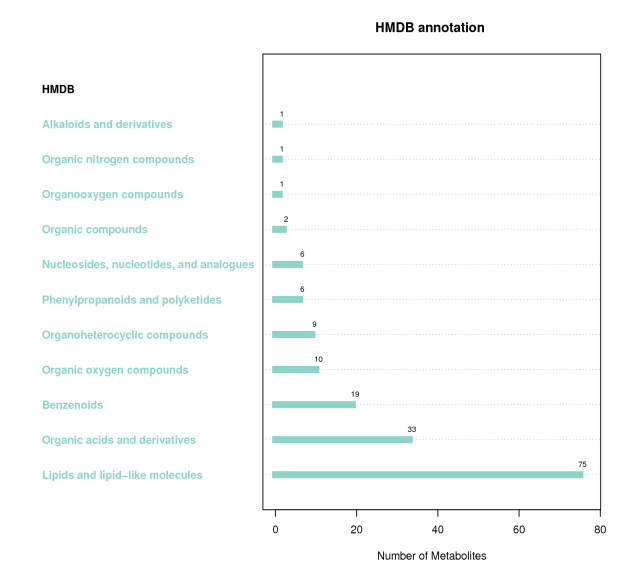

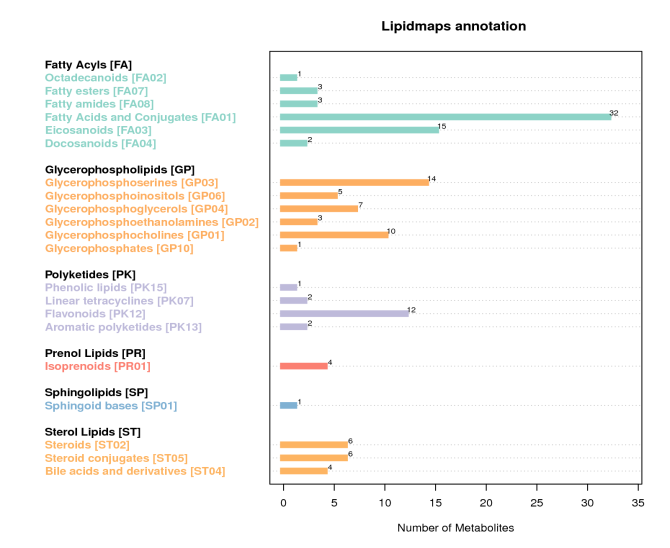

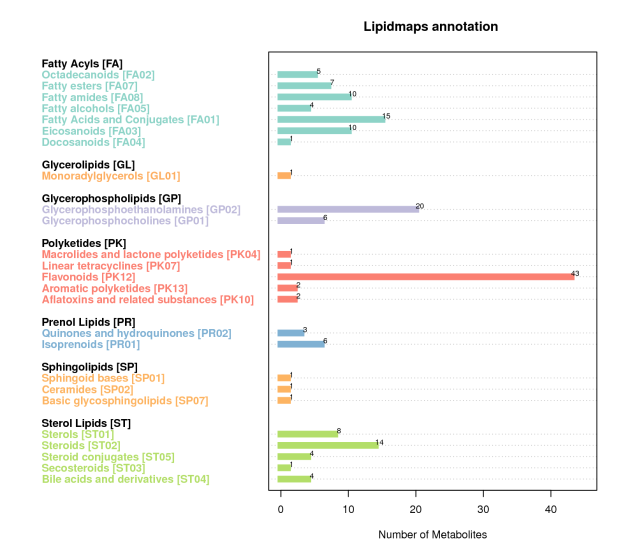


**Fig. S6** Fecal metabolite classification annotation results in the Main_Class of the LIPID MAPS database (8 lipid categories): (A) negative ion mode (neg.); (B) positive ion mode (pos.).

**A**

**B**

**Fig. S7** Serum metabolite classification annotation results in the Main_Class of the LIPID MAPS database (8 lipid categories): (A) negative ion mode (neg.); (B) positive ion mode (pos.).

**A**

**B**


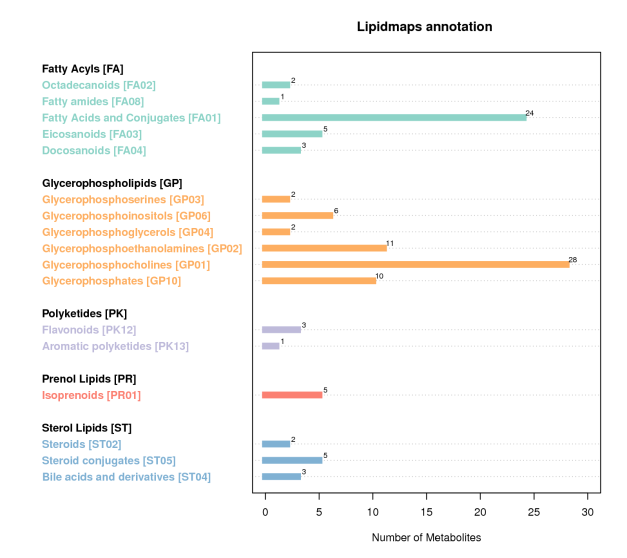

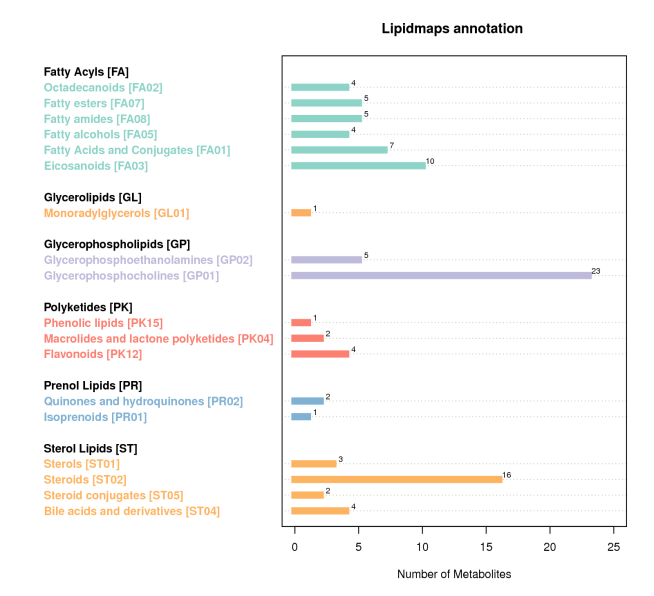


**Fig. S8** (A) and (B) are the Scatter plot of PLS-DA scores in positive and negative ion mode for serum metabolites, respectively. The abscissa is the score of the sample on the first principal component, and the ordinate is the score of the sample on the second principal component. R2Y represents the interpretation rate of the model, Q2Y is used to evaluate the predictive ability of the PLS-DA model, and when R2Y is greater than Q2Y, the model is well established. (D) and (E) are bubble plots of serum differential metabolite pathway enrichment in positive and negative ion modes, respectively. The abscissa in the figure is the number of differential metabolites in the corresponding metabolic pathway/the total number of metabolites identified in the pathway. The larger the value, the higher the enrichment of differential metabolites in the pathway. The color of the dots represents the p-value of the hypergeometric test, and the smaller the value, the greater the reliability of the test. The size of the dots represents the number of differential metabolites in the corresponding pathway.

**A**

**B**


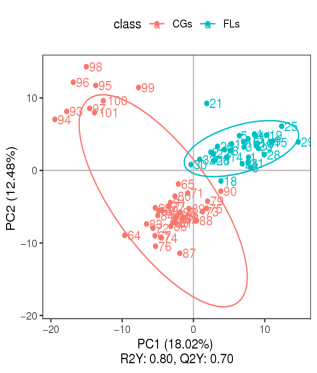

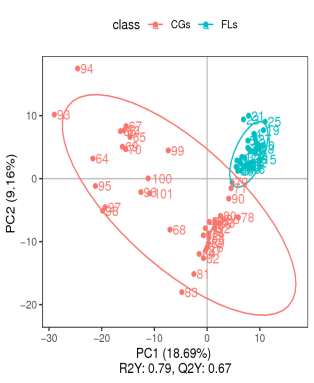


**C**

**D**


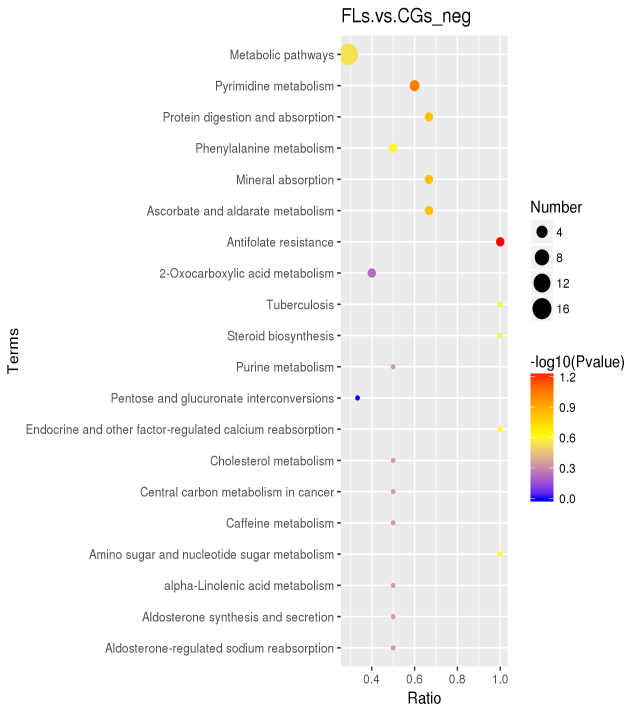

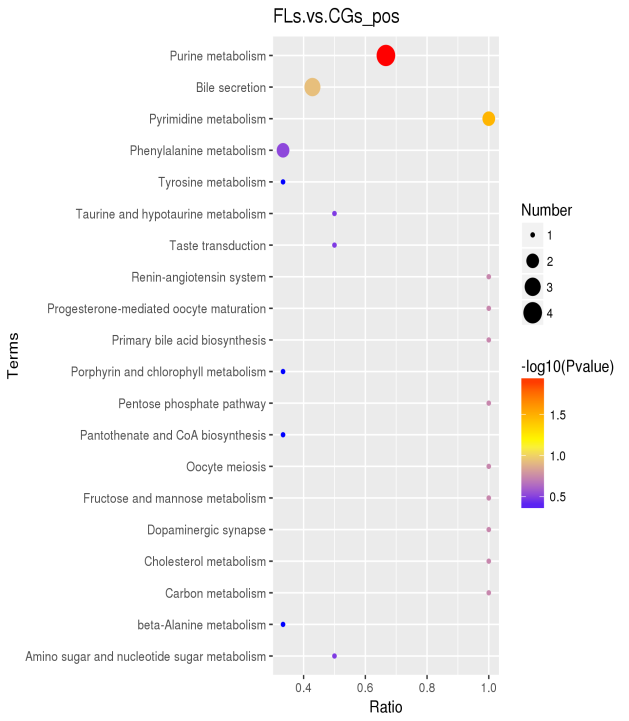

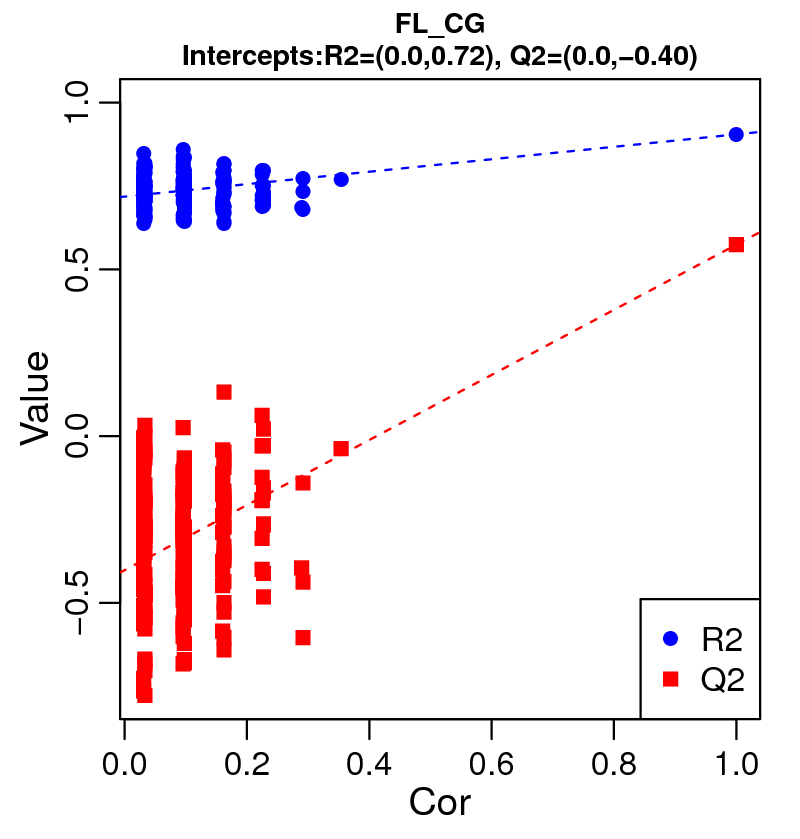

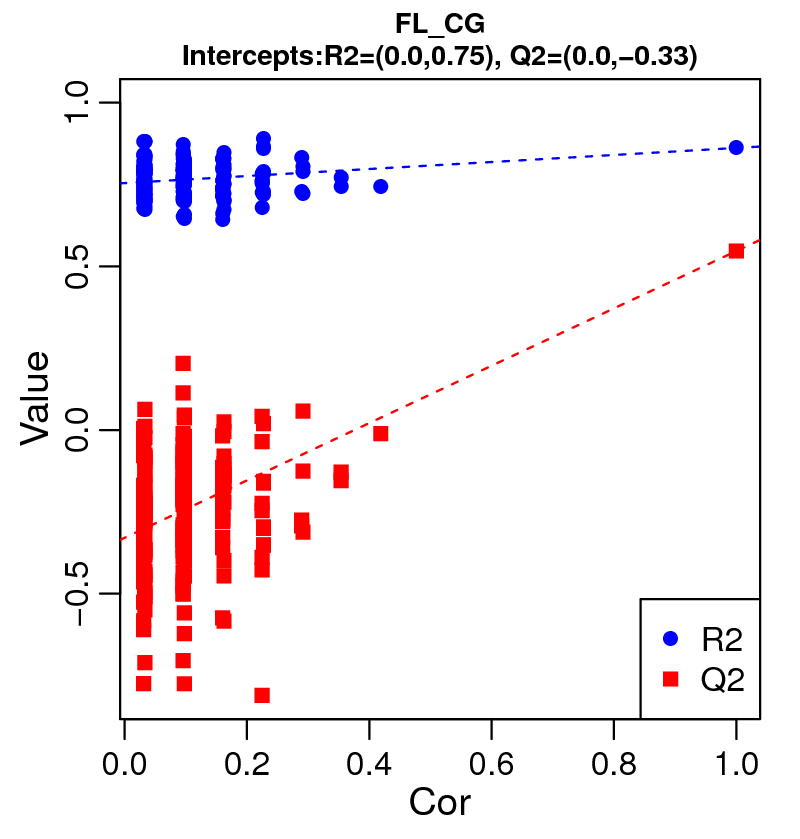

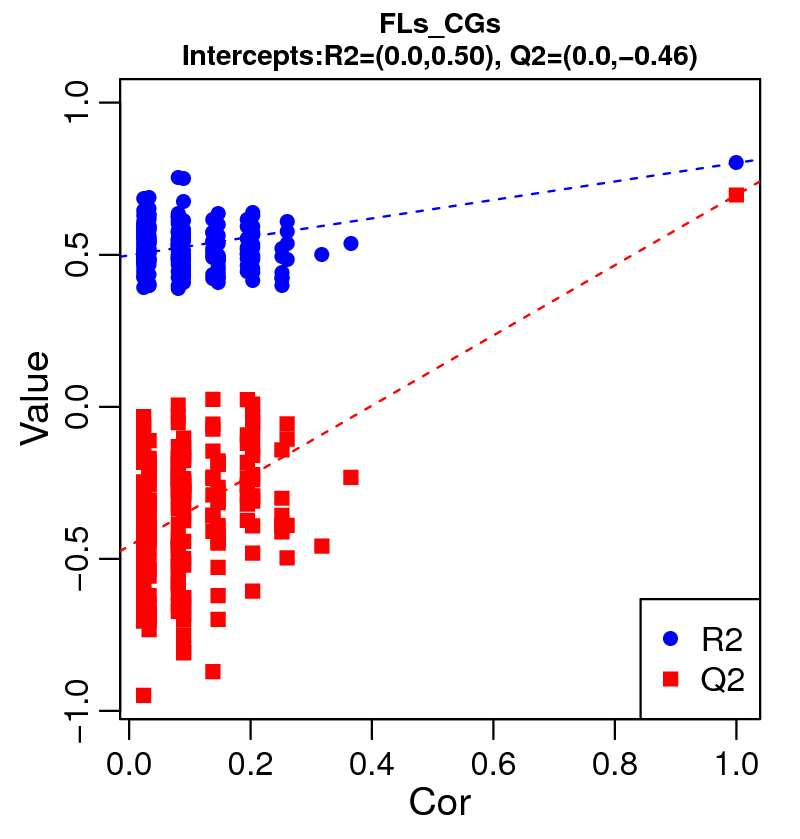

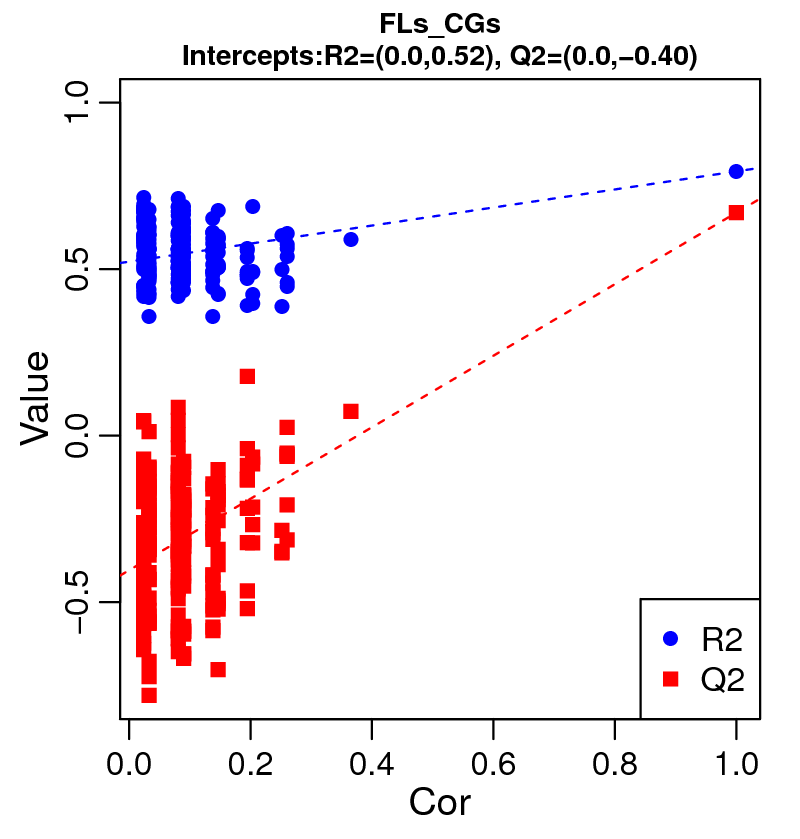


**A**

**B**

**C**

**D**

**Fig.S9** PLS-DA ranking verification graphs. (A): negative ion mode of fecal metabolites; (B) positive ion mode of fecal metabolites; (C): negative ion mode of serum metabolites; (D) positive ion mode of serum metabolites. When R2 was greater than Q2 and the Q2 regression line and the Y-axis intercept were less than 0, it could indicate that the model was not "overfitting".


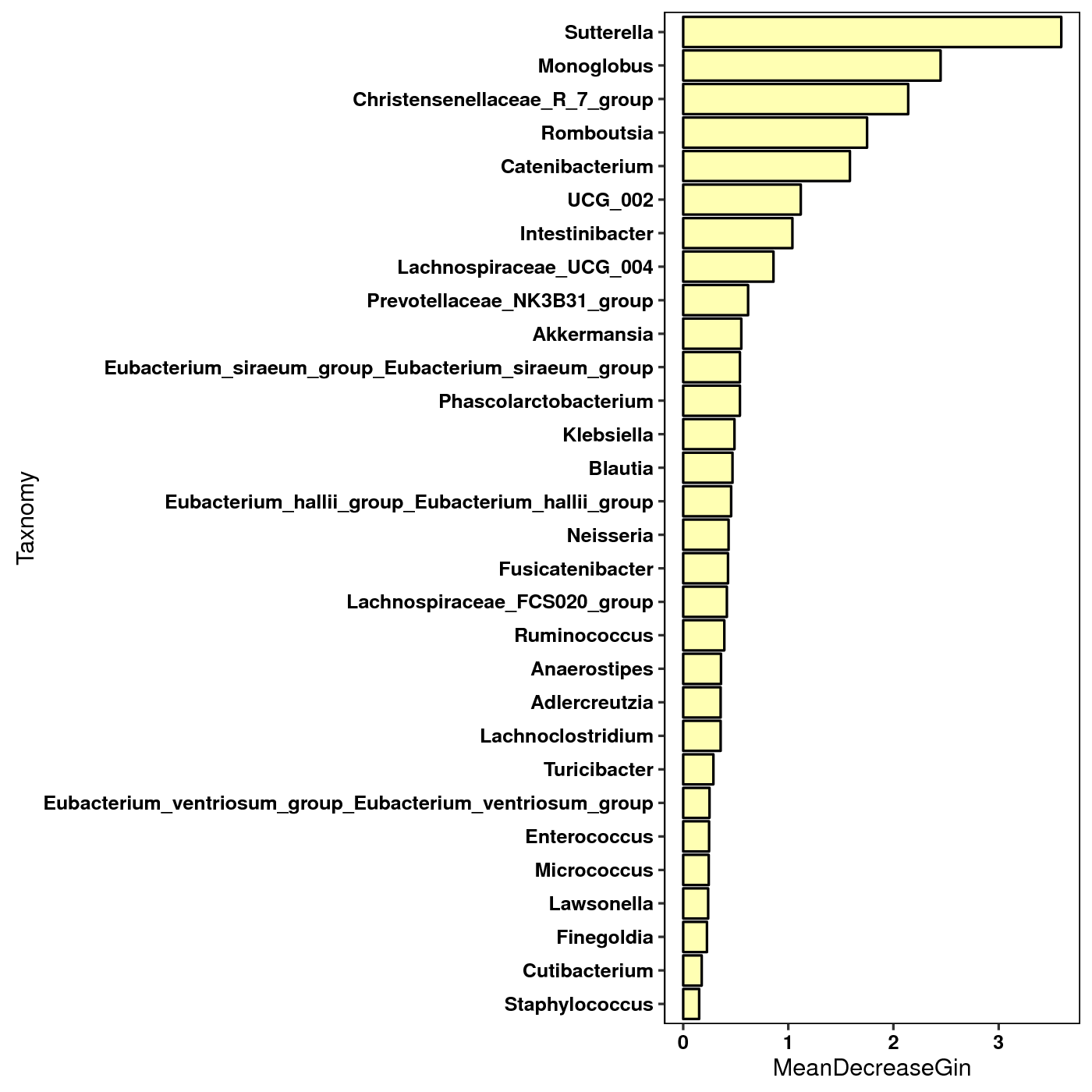

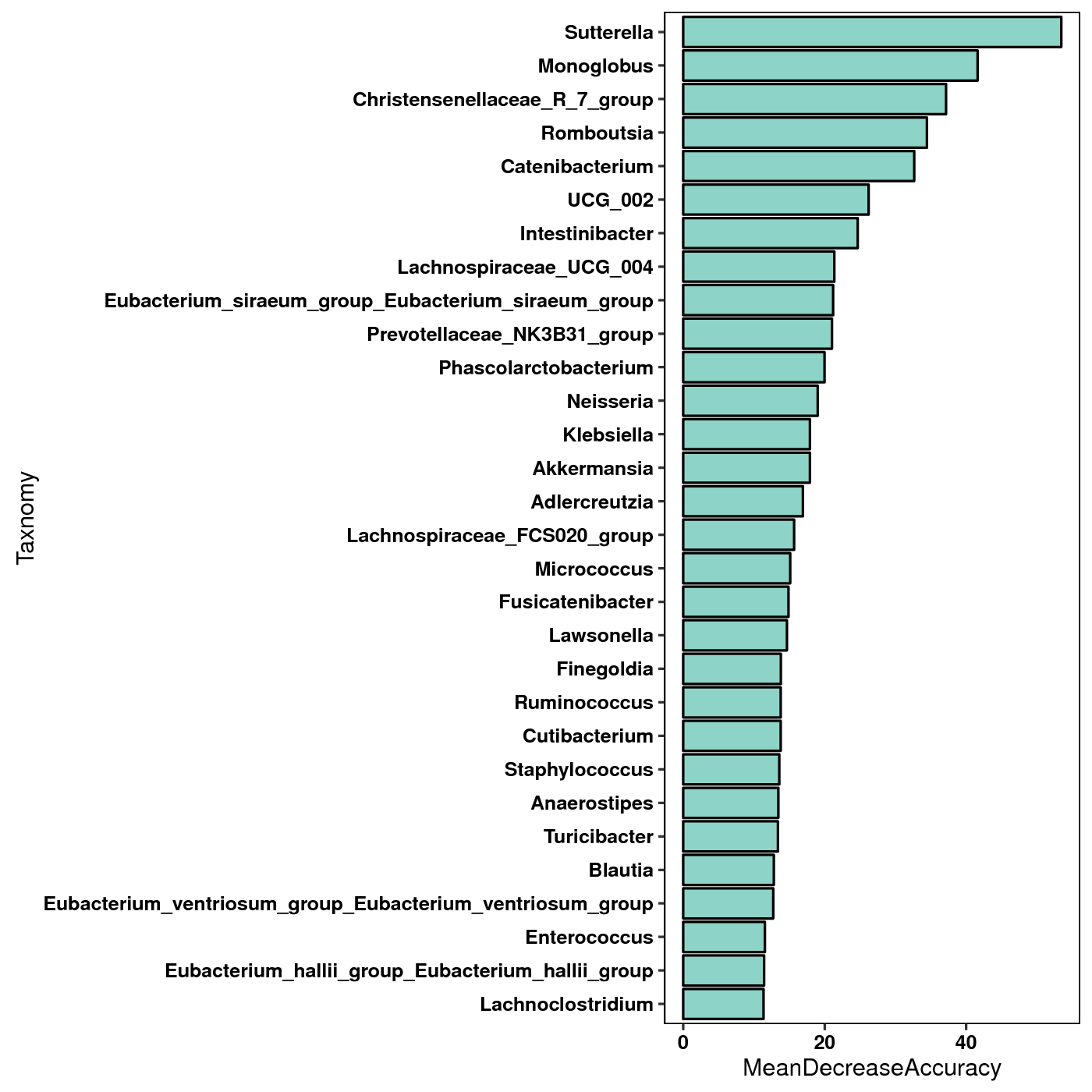


**A**

**B**

**C**

**Fig. S10** (A) and (B) The variable importance ranking diagram of random forest model based on genus-level taxons; (C) The ROC curve of the test set; (D) The ROC curve of the validation set.


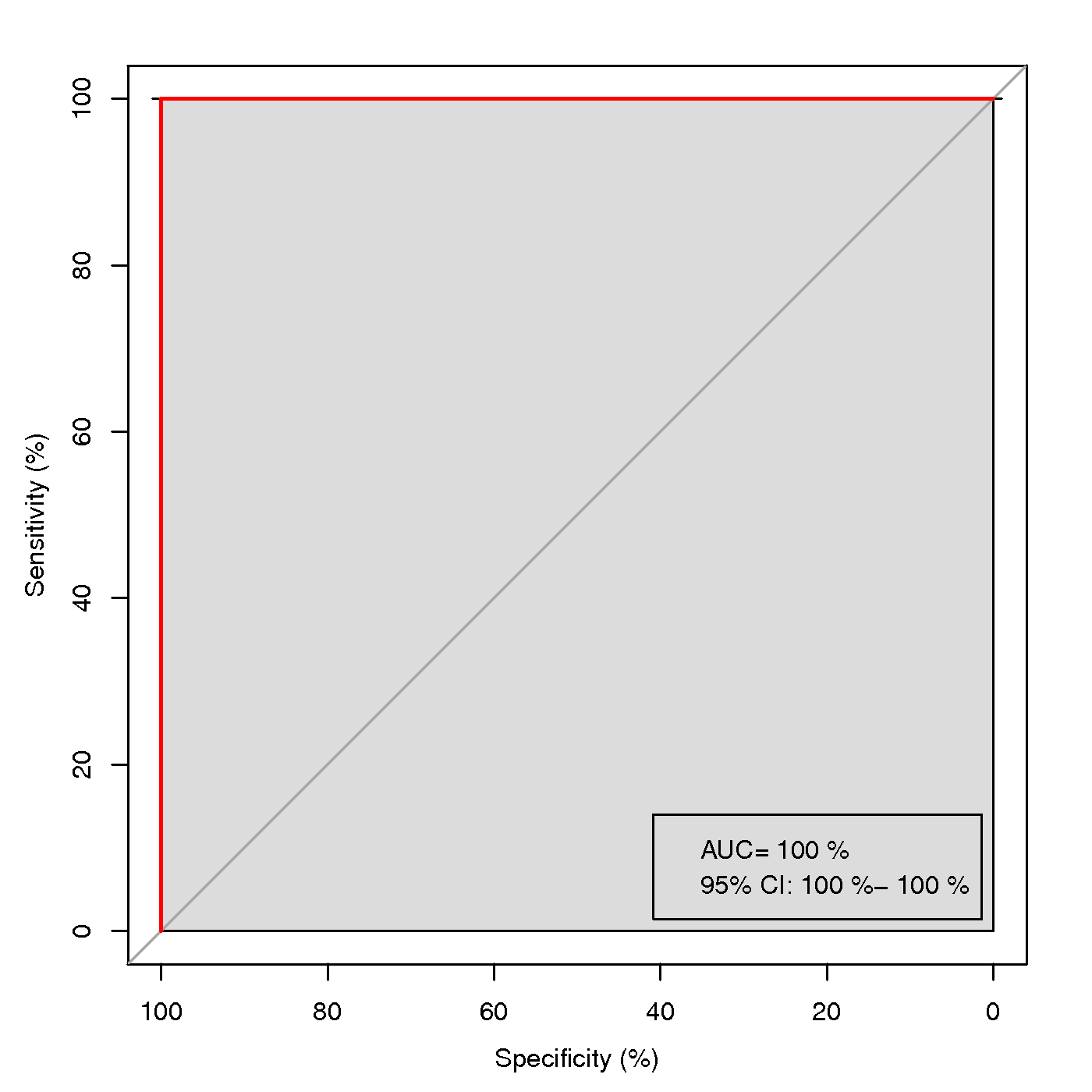

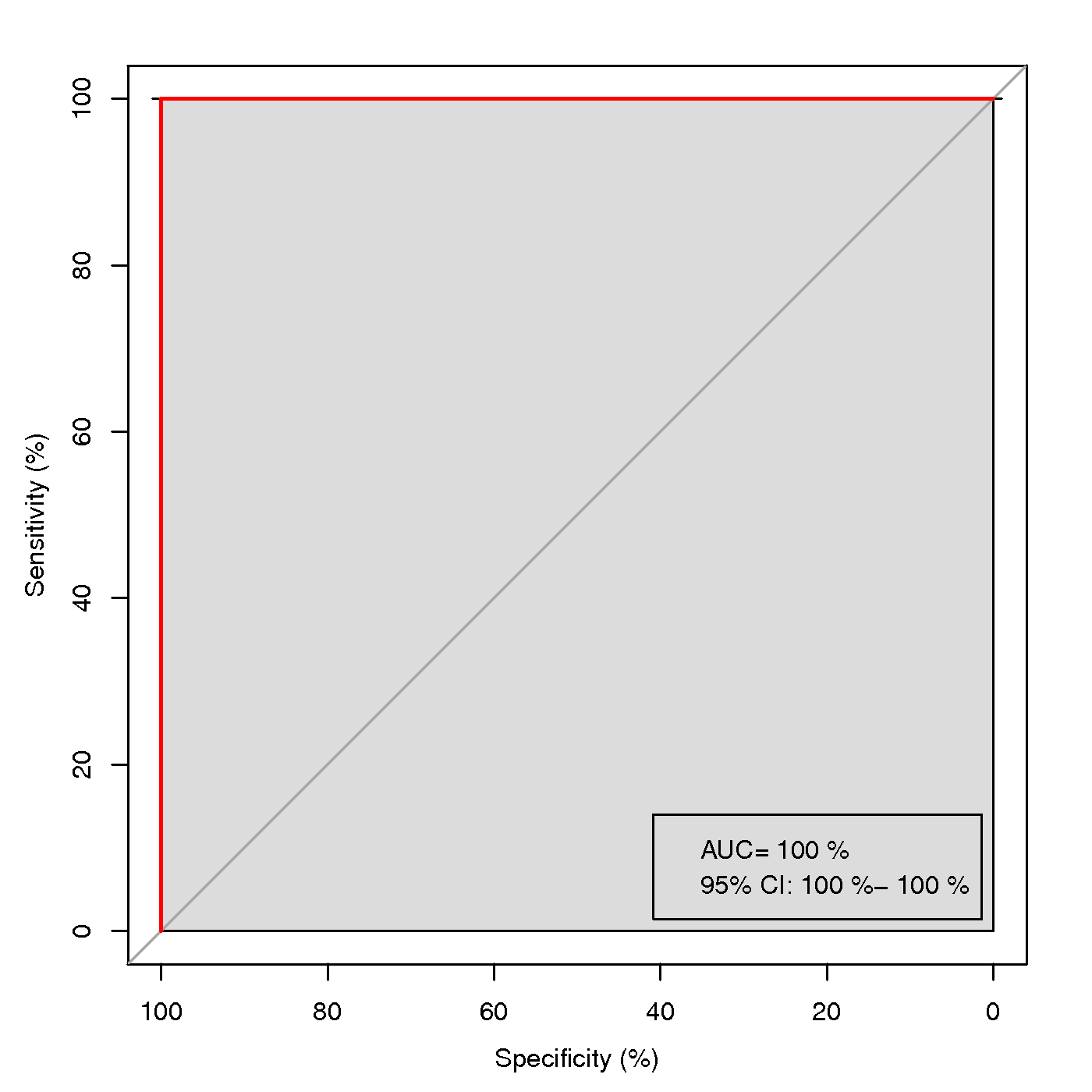


**D**


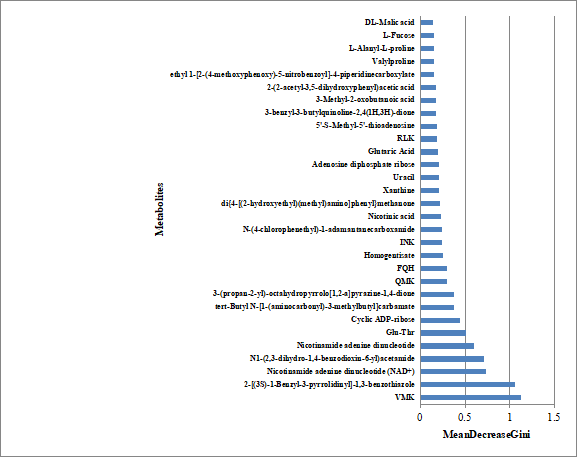

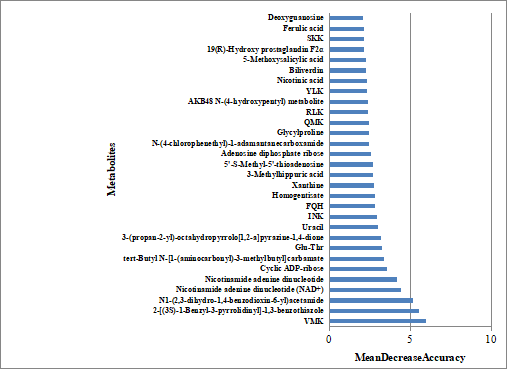


**A**

**B**

**C**

**Fig. S11** (A) and (B) The variable importance ranking plots of random forest model based on fecal metabolites; (C) The ROC curve of the test set; (D) The ROC curve of the validation set.

**D**


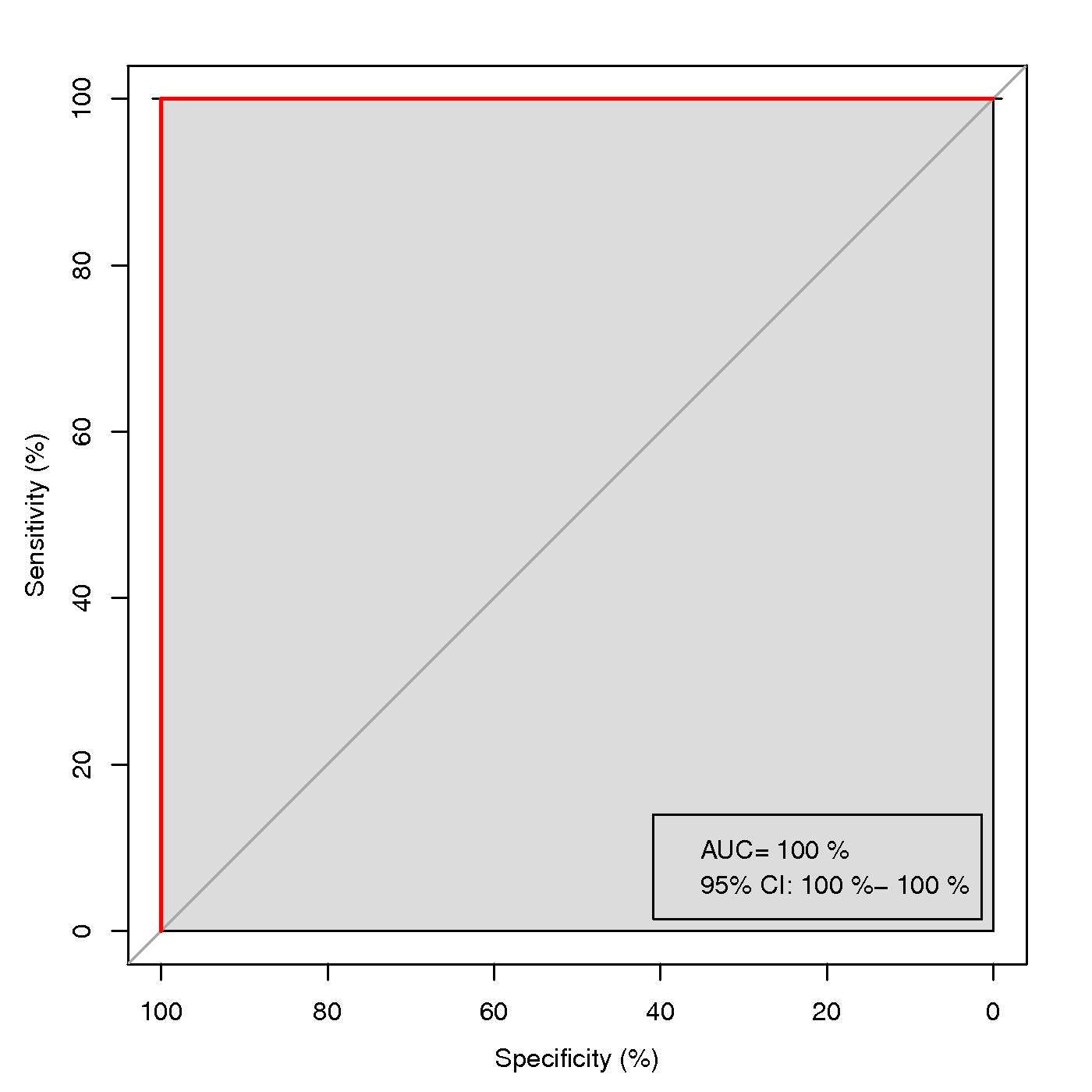

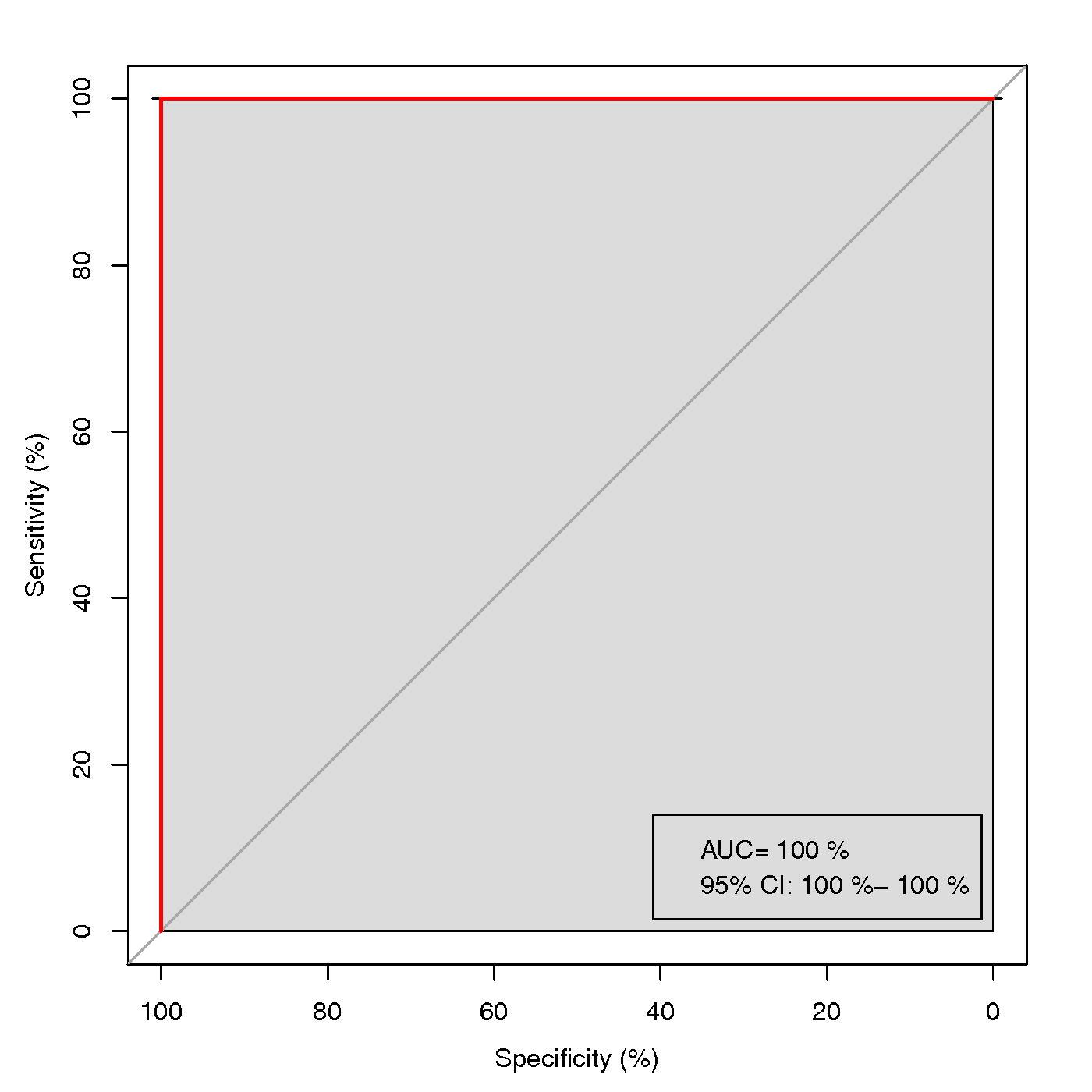

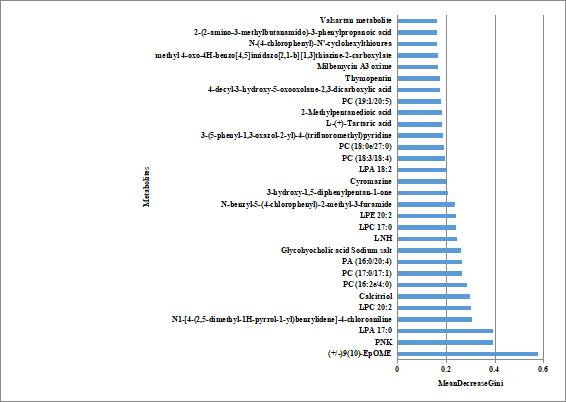

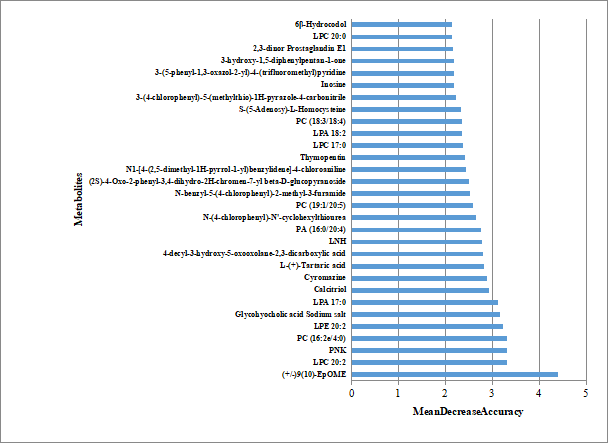


**A**

**B**

**C**

**Fig. S12** (A) and (B) The variable importance ranking plots of random forest model based on serum metabolites; (C) The ROC curve of the test set; (D) The ROC curve of the validation set.

**D**


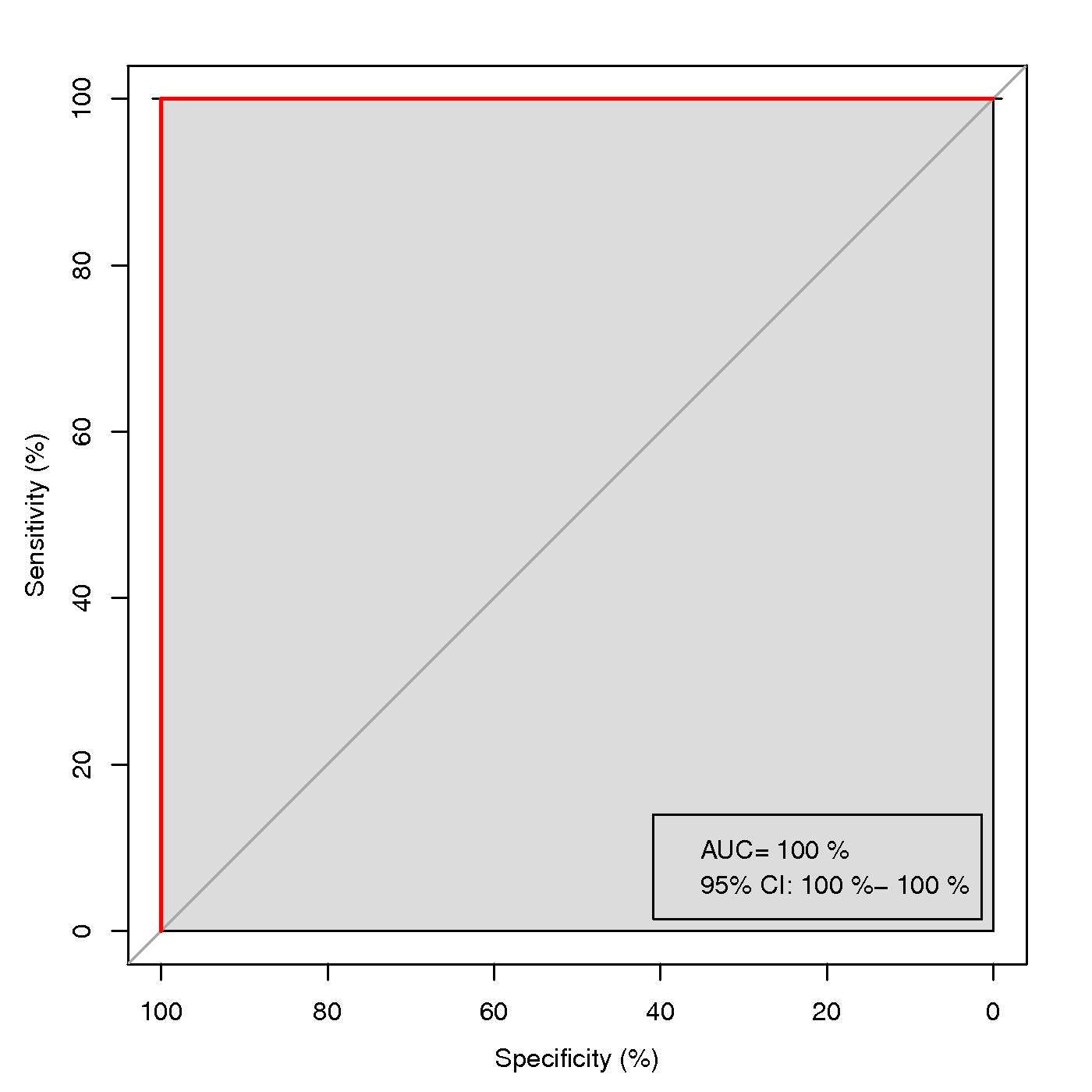

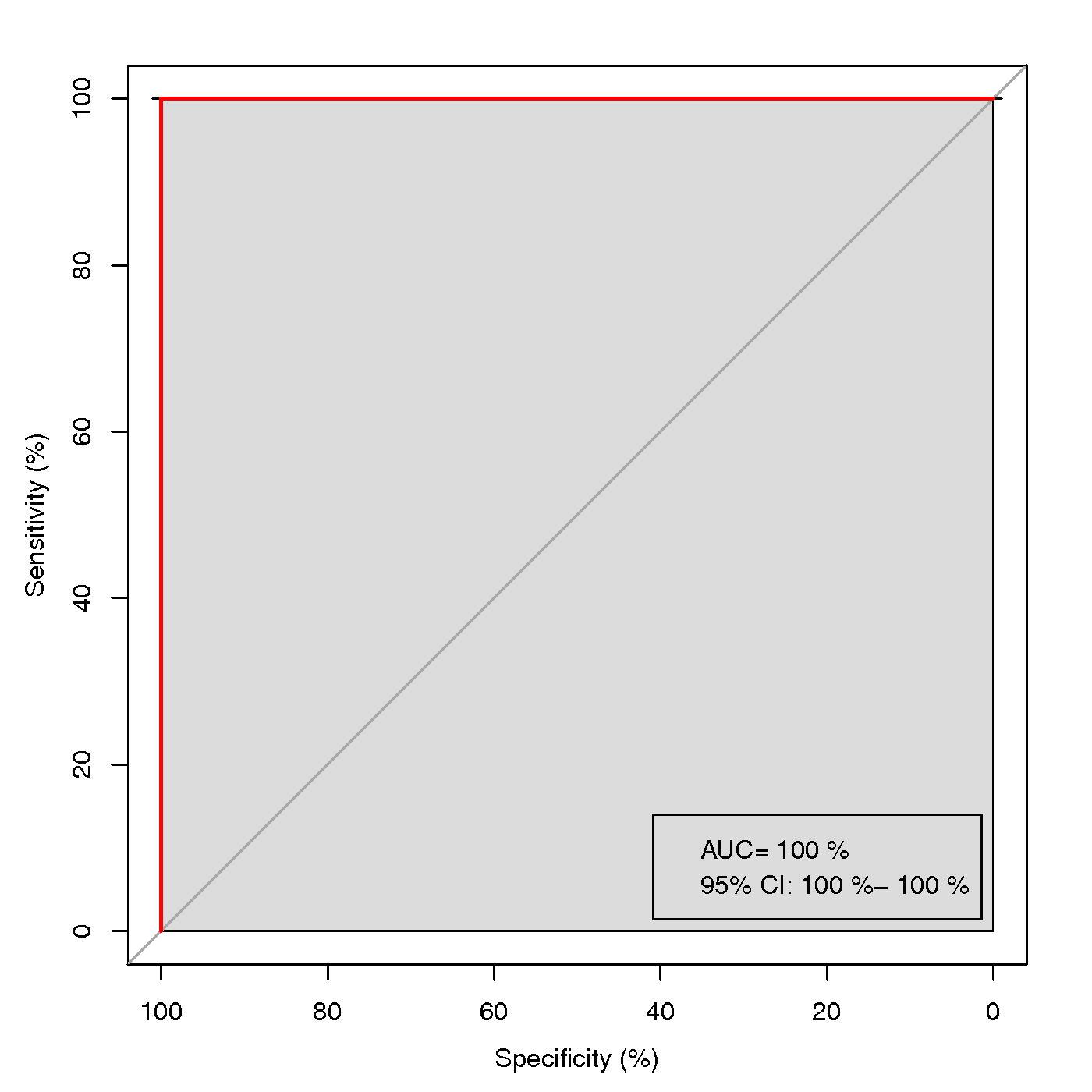


1. **Supplementary Tables**

**Table S1. Comparison of clinical characteristics and laboratory test indexes between MAFLD group and control group**

| Variables | Control Group  N=30 | MAFLD Group  N=32 | *p* |
| --- | --- | --- | --- |
| Age (year) | 35.33 (32.50-51.25) | 38.50 (33.00-51.75) | 0.577 |
| Gender (male/ female) | 18/12 | 26/6 | 0.065 |
| Height (cm) | 165.56 ± 7.38 | 168.89 ± 7.59 | 0.093 |
| Weight (Kg) | 65.56 ± 11.38 | 74.75 ± 11.73 | 0.003 |
| BMI（kg/cm^2^） | 23.83 ± 3.28 | 26.21 ± 3.80 | 0.013 |
| TBIL (umol/L) | 13.30 (9.10-19.38) | 12.9 (10.88-17.03) | 0.627 |
| DBIL (umol/L) | 3.45 (2.48-4.85) | 3.45 (2.83-4.33) | 0.849 |
| IBIL (umol/L) | 9.65 (6.60-13.68) | 9.85 (8.43-12.68) | 0.310 |
| ALT (IU/L) | 18.00 (13.00-26.50) | 58.5 (47.25-100.00) | <0.001 |
| AST(IU/L) | 19.5 (17.75-22.50) | 31.5 (26.00-49.50) | <0.001 |
| ALP(IU/L) | 64.6 ± 16.72 | 81.94 ± 19.15 | <0.001 |
| GGT(IU/L) | 13.50 (10.75-28.75) | 55.00 (32.25-98.00) | <0.001 |
| TP(g/L) | 74.40 (72.08-78.08) | 74.56(72.70-77.35) | 0.714 |
| ALB(g/L) | 46.65 (45.30-47.90) | 49.20(45.83-50.25) | 0.011 |
| GLB(g/L) | 28.21 ± 2.92 | 27.15 ± 3.57 | 0.204 |
| Fasting blood glucose (mmol/L) | 4.98 (4.75-5.13) | 5.16 (4.70-6.13) | 0.049 |
| UREA (mmol/L) | 5.05(4.13-5.63) | 4.65 (3.90-5.50) | 0.394 |
| CREA (umol/L) | 74.5 (62.75-89.00) | 78.00 (70.00-90.75) | 0.195 |
| eGFR(ml/min/1.73m^2^) | 98.12 ± 19.74 | 97.36 ± 16.06 | 0.880 |
| URIC (umol/L) | 297.00 (248.25-377.00) | 417(344.00-468.00) | <0.001 |
| TG (mmol/L) | 1.00(0.81-1.39) | 1.65(1.38-2.44) | <0.001 |
| CHOL (mmol/L) | 4.97 ± 0.86 | 4.82 ± 0.90 | 0.486 |
| HDL-C (mmol/L) | 1.55 ± 0.33 | 1.07 ± 0.20 | <0.001 |
| LDL-C (mmol/L) | 2.96 ± 0.77 | 2.99 ± 0.73 | 0.873 |
| TSH (mU/L) | 2.37 (1.58-3.06) | 2.51 (1.68-4.01) | 0.438 |
| FT3(pmol/L) | 5.40 ± 1.45 | 5.07 ± 0.56 | 0.290 |
| FT4(pmol/L) | 16.25(14.65-17.7) | 15.65(14.38-17.40) | 0.478 |
| FIB-4^*^ | 0.65 (0.33-0.85) | 1.25 (0.70-1.92) | 0.09 |

Note: Fibrosis-4 index = (Age(year)×AST(U/L)/(PLT(×10^9^/L)×ALT(U/L)^1/2^)

**Table S2. Common differential metabolites in serum and feces**

| Name | **Other_name (Kegg_name)** | **SubClass (HMDB)** | **SUB_CLASS (Lipidmaps)** | **Fecal** | **Serum** |
| --- | --- | --- | --- | --- | --- |
| Hypoxanthine | Hypoxanthine; Purine-6-ol | Purines and purine derivatives | -- | down | down |
| Propionylcarnitine | -- | Fatty acid esters | Fatty acyl carnitines [FA0707] | down | down |
| 2-(2-amino-3-methylbutanamido)-3-phenylpropanoic acid | -- | -- | -- | down | down |
| Tyrosylalanine | -- | Amino acids, peptides, and analogues | -- | down | down |
| 5,6-diphenyl-3-(2-pyridyl)-1,2,4-triazine | -- | -- | -- | down | down |
| 3-(5-phenyl-1,3-oxazol-2-yl)-4-(trifluoromethyl)pyridine | -- | -- | -- | down | down |
| AKB48 N-(4-fluorobenzyl) analog | -- | -- | -- | down | down |
| 2-[5-(2-hydroxypropyl)oxolan-2-yl]propanoic acid | -- | -- | -- | down | down |
| PC (16:2e/2:0) | -- | -- | -- | up | down |
| Hesperetin | Hesperetin; 3',5,7-Trihydroxy-4'-methoxyflavanone | O-methylated flavonoids | Flavanones [PK1214] | down | down |
| 4-acetyl-4-(ethoxycarbonyl)heptanedioic acid | -- | -- | -- | down | down |
| PC (14:0e/3:0) | -- | -- | -- | up | down |
| N-(2-morpholinophenyl)-2-furamide | -- | -- | -- | down | down |
| 5,5-dimethyl-3-morpholinocyclohex-2-en-1-one | -- | -- | -- | down | down |
| 1H-indene-3-carboxamide | -- | -- | -- | down | down |
| 1-(4-benzylpiperazino)-2-(pyridin-2-ylamino)propan-1-one | -- | -- | -- | down | down |
| Methionine | L-Methionine; Methionine; L-2-Amino-4methylthiobutyric acid | Amino acids, peptides, and analogues | -- | down | down |
| LPC 18:0 | -- | -- | Monoacylglyce-rophosphochol-ines [GP0105] | up | down |
| Gamma-Glu-Leu | -- | Amino acids, peptides, and analogues | -- | down | down |
| Propylparaben | -- | Benzoic acids and derivatives | -- | down | down |
| LPC 16:0 | -- | -- | Monoacylglyce-rophosphochol-ines [GP0105] | up | up |
| Neohesperidin | Neohesperidin; Hesperetin 7-O-neohesperidoside | Flavonoid glycosides | Flavanones [PK1214] | down | down |

**Table S3. Correlation analysis results of the common differential metabolites and fecal differential bacteria**

|  | differential bacteria | | differential metabolites | rho | P |
| --- | --- | --- | --- | --- | --- |
| faeces | Agathobacter | 2-(2-amino-3-methylbutanamido)-3-phenylpropanoic acid | | 0.569 | <0.05 |
|  | Subdoligranulum | 2-[5-(2-hydroxypropyl)oxolan-2-yl]propanoic acid | | 0.544 | <0.05 |
|  | Christensenellaceae_R-7_group | 2-(2-amino-3-methylbutanamido)-3-phenylpropanoic acid | | 0.538 | <0.05 |
|  |  | 3-(5-phenyl-1,3-oxazol-2-yl)-4-(trifluoromethyl)pyridine | | 0.626 | <0.05 |
|  |  | 2-[5-(2-hydroxypropyl)oxolan-2-yl]propanoic acid | | 0.737 | <0.05 |
|  |  | 4-acetyl-4-(ethoxycarbonyl)heptanedioic acid | | 0.629 | <0.05 |
|  |  | N-(2-morpholinophenyl)-2-furamide | | 0.686 | <0.05 |
|  | Ruminococcus | 2-(2-amino-3-methylbutanamido)-3-phenylpropanoic acid | | 0.502 | <0.05 |
|  | UCG-002 | 3-(5-phenyl-1,3-oxazol-2-yl)-4-(trifluoromethyl)pyridine | | 0.755 | <0.05 |
|  |  | 2-[5-(2-hydroxypropyl)oxolan-2-yl]propanoic acid | | 0.83 | <0.05 |
|  |  | 4-acetyl-4-(ethoxycarbonyl)heptanedioic acid | | 0.545 | <0.05 |
|  |  | N-(2-morpholinophenyl)-2-furamide | | 0.643 | <0.05 |
|  | Monoglobus | 2-(2-amino-3-methylbutanamido)-3-phenylpropanoic acid | | 0.599 | <0.05 |
|  | Adlercreutzia | 4-acetyl-4-(ethoxycarbonyl)heptanedioic acid | | 0.64 | <0.05 |
|  | NK4A214_group | 4-acetyl-4-(ethoxycarbonyl)heptanedioic acid | | 0.831 | <0.05 |
|  |  | N-(2-morpholinophenyl)-2-furamide | | 0.772 | <0.05 |
|  |  | 5,5-dimethyl-3-morpholinocyclohex-2-en-1-one | | 0.55 | <0.05 |
|  | Lachnospiraceae_ND3007_group | 2-(2-amino-3-methylbutanamido)-3-phenylpropanoic acid | | 0.593 | <0.05 |
|  | UCG-005 | 2-[5-(2-hydroxypropyl)oxolan-2-yl]propanoic acid | | 0.54 | <0.05 |
|  |  | 4-acetyl-4-(ethoxycarbonyl)heptanedioic acid | | 0.597 | <0.05 |
|  |  | N-(2-morpholinophenyl)-2-furamide | | 0.558 | <0.05 |
|  |  | 5,5-dimethyl-3-morpholinocyclohex-2-en-1-one | | 0.551 | <0.05 |
|  | Lachnospiraceae_FCS020_group | 1-(4-benzylpiperazino)-2-(pyridin-2-ylamino)propan-1-one | | 0.531 | <0.05 |
|  | Sellimonas | N-(2-morpholinophenyl)-2-furamide | | 0.517 | <0.05 |
|  | Family_XIII_AD3011_group | 4-acetyl-4-(ethoxycarbonyl)heptanedioic acid | | 0.857 | <0.05 |
|  |  | N-(2-morpholinophenyl)-2-furamide | | 0.84 | <0.05 |
|  | Family_XIII_UCG-001 | 2-[5-(2-hydroxypropyl)oxolan-2-yl]propanoic acid | | 0.686 | <0.05 |
|  |  | N-(2-morpholinophenyl)-2-furamide | | 0.574 | <0.05 |
|  | GCA-900066575 | 3-(5-phenyl-1,3-oxazol-2-yl)-4-(trifluoromethyl)pyridine | | 0.505 | <0.05 |
|  |  | 2-[5-(2-hydroxypropyl)oxolan-2-yl]propanoic acid | | 0.526 | <0.05 |
|  |  | 4-acetyl-4-(ethoxycarbonyl)heptanedioic acid | | 0.513 | <0.05 |
|  | Papillibacter | 4-acetyl-4-(ethoxycarbonyl)heptanedioic acid | | 0.570 | <0.05 |
|  |  | N-(2-morpholinophenyl)-2-furamide | | 0.628 | <0.05 |
|  |  | 5,5-dimethyl-3-morpholinocyclohex-2-en-1-one | | 0.539 | <0.05 |
| serum | Erysipelotrichaceae_UCG-003 | N-(2-morpholinophenyl)-2-furamide | | 0.654 | <0.05 |
|  |  | 2-[5-(2-hydroxypropyl)oxolan-2-yl]propanoic acid | | 0.631 | <0.05 |
|  |  | 2-(2-amino-3-methylbutanamido)-3-phenylpropanoic acid | | 0.642 | <0.05 |
|  | Christensenellaceae_R-7_group | LPC 18:0 | | 0.601 | <0.05 |
|  | UCG-002 | LPC 18:0 | | 0.612 | <0.05 |
|  | Erysipelotrichaceae_UCG-003 | Propylparaben | | 0.555 | <0.05 |
|  | Peptoniphilus | Neohesperidin | | 0.658 | <0.05 |
|  | Phycicoccus | Neohesperidin | | 0.558 | <0.05 |
|  | Stomatobaculum | Neohesperidin | | 0.551 | <0.05 |

Note: LPC: lysophosphatidylcholine

**Table S4. Correlation analysis results of the differential bile acids and derivatives and fecal differential bacteria**

|  | differential bacteria | | differential metabolites | rho | *P* |
| --- | --- | --- | --- | --- | --- |
| faeces | Subdoligranulum | Taurodeoxycholic Acid | | 0.505 | <0.05 |
|  | Prevotellaceae_NK3B31_group | Allolithocholic acid | | 0.723 | <0.05 |
|  |  | Dehydrocholic acid | | 0.589 | <0.05 |
|  | unidentified_Ruminococcaceae | Allolithocholic acid | | 0.797 | <0.05 |
|  |  | Dehydrocholic acid | | 0.523 | <0.05 |
|  | Parvibacter | Allolithocholic acid | | 0.504 | <0.05 |
| serum | Erysipelotrichaceae_UCG-003 | Taurocholic acid | | 0.563 | <0.05 |

**Table S5. Correlation analysis results of the differential lipids and lipid-related molecules in serum and fecal differential bacteria**

| differential bacteria | differential metabolites | | rho | *P* |
| --- | --- | --- | --- | --- |
| Parvibacter | | PG (18:1/18:2) | 0.638 | <0.05 |
| Family_XIII_UCG-001 | | PG (18:1/18:2) | 0.519 | <0.05 |
| Gordonibacter | | Taurochenodeoxycholic Acid (sodium salt) | 0.521 | <0.05 |
| Intestinibacter | | 2-Ketohexanoic acid | 0.557 | <0.05 |
| Lachnospiraceae_FCS020_group | | Neohesperidin | 0.503 | <0.05 |
| Peptoniphilus | | UDP-D-glucuronate | 0.658 | <0.05 |
| Phycicoccus | | UDP-D-glucuronate | 0.557 | <0.05 |
| Stomatobaculum | | UDP-D-glucuronate | 0.552 | <0.05 |
|  | | L-Leucyl-L-alanine Hydrate | 0.566 | <0.05 |
| Erysipelatoclostridium | | Gamma-Glu-Leu | 0.566 | <0.05 |
| Monoglobus | | Gamma-Glu-Leu | 0.540 | <0.05 |
| Lautropia | | L-Leucyl-L-alanine Hydrate | 0.568 | <0.05 |
| Lachnoanaerobaculum | | L-Leucyl-L-alanine Hydrate | 0.889 | <0.05 |
| Erysipelotrichaceae_UCG-003 | | Tylosin | 0.658 | <0.05 |
|  | | Taurocholic acid | 0.552 | <0.05 |
|  | | Palmitoyl ethanolamide | 0.553 | <0.05 |
| Fusicatenibacter | | 6-Keto-prostaglandin f1alpha | 0.743 | <0.05 |
| Blautia | | 6-Keto-prostaglandin f1alpha | 0.611 | <0.05 |
| Anaerostipes | | 6-Keto-prostaglandin f1alpha | 0.730 | <0.05 |
| Christensenellaceae_R-7_group | | Progesterone | 0.508 | <0.05 |
| Dorea | | 6-Keto-prostaglandin f1alpha | 0.507 | <0.05 |
| [Eubacterium]_ventriosum_group | | Palmitoyl ethanolamide | 0.533 | <0.05 |
| Turicibacter | | Tomatidine | 0.646 | <0.05 |
| Weissella | | PC (17:1/17:1) | 0.538 | <0.05 |
| [Eubacterium]_fissicatena_group | | Tylosin | 0.501 | <0.05 |
|  | | Palmitoyl ethanolamide | 0.590 | <0.05 |
| Family_XIII_AD3011_group | | Progesterone | 0.605 | <0.05 |
